# Anneal-free ultra-low loss silicon nitride integrated photonics

**DOI:** 10.1038/s41377-024-01503-4

**Published:** 2024-07-08

**Authors:** Debapam Bose, Mark W. Harrington, Andrei Isichenko, Kaikai Liu, Jiawei Wang, Nitesh Chauhan, Zachary L. Newman, Daniel J. Blumenthal

**Affiliations:** 1 Department of Electrical and Computer Engineering, University of California Santa Barbara, Santa Barbara, CA 93106 USA; 2 Octave Photonics, Louisville, CO 80027 USA

**Keywords:** Optical materials and structures, Supercontinuum generation, Optical metrology

## Abstract

Heterogeneous and monolithic integration of the versatile low-loss silicon nitride platform with low-temperature materials such as silicon electronics and photonics, III–V compound semiconductors, lithium niobate, organics, and glasses has been inhibited by the need for high-temperature annealing as well as the need for different process flows for thin and thick waveguides. New techniques are needed to maintain the state-of-the-art losses, nonlinear properties, and CMOS-compatible processes while enabling this next generation of 3D silicon nitride integration. We report a significant advance in silicon nitride integrated photonics, demonstrating the lowest losses to date for an anneal-free process at a maximum temperature 250 °C, with the same deuterated silane based fabrication flow, for nitride and oxide, for an order of magnitude range in nitride thickness without requiring stress mitigation or polishing. We report record low anneal-free losses for both nitride core and oxide cladding, enabling 1.77 dB m^-1^ loss and 14.9 million Q for 80 nm nitride core waveguides, more than half an order magnitude lower loss than previously reported sub 300 °C process. For 800 nm-thick nitride, we achieve as good as 8.66 dB m^−1^ loss and 4.03 million Q, the highest reported Q for a low temperature processed resonator with equivalent device area, with a median of loss and Q of 13.9 dB m^−1^ and 2.59 million each respectively. We demonstrate laser stabilization with over 4 orders of magnitude frequency noise reduction using a thin nitride reference cavity, and using a thick nitride micro-resonator we demonstrate OPO, over two octave supercontinuum generation, and four-wave mixing and parametric gain with the lowest reported optical parametric oscillation threshold per unit resonator length. These results represent a significant step towards a uniform ultra-low loss silicon nitride homogeneous and heterogeneous platform for both thin and thick waveguides capable of linear and nonlinear photonic circuits and integration with low-temperature materials and processes.

## Abstract (web summary)

We demonstrate for the first time, a uniform low temperature (<250 °C) process for fabricating both high-confinement thick and low-confinement thin ultra-low loss Silicon nitride waveguides.

## Graphical abstract

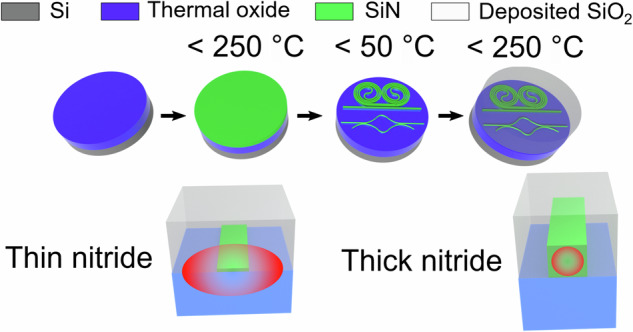

## Introduction

Ultra-low loss silicon nitride photonic integrated circuits^1^ (PICs) have the potential to reduce the size, weight, and cost, and improve the reliability of a wide range of applications spanning the visible to infrared. These applications include quantum computing and sensing^2–5^, atomic clocks^6,7^, atomic navigation^8^, metrology^9^, and fiber optic communications^10^, as well as new portable applications^11^. In addition to replacing costly systems such as lasers and optical frequency combs that are relegated to bulky table-top systems, there is the potential to improve the performance of precision sciences, such as reducing laser frequency noise, which is important for the manipulation and interrogation of atom, ions, and qubits^12,13^. The silicon nitride integration platform has enabled a wide range of waveguide and device designs, from thin nitride waveguides that support ultra-low loss dilute optical modes to thick nitride waveguides that are strongly confining and enhance optical nonlinearities. By varying waveguide parameters, such as nitride core thickness and width, it is possible to design characteristics such as loss, dispersion, nonlinearity, and device footprint^14–16^. Leveraging the properties of both thin weakly confining and thick strongly confining waveguides, this platform enables the designer to realize a wide range of components and functions, including ultra-low linewidth lasers^17–21^, optical frequency combs^22^, optical modulators^23,24^, tunable lasers and filters^25,26^, and atom and ion beam emitters^2,27–29^.

Yet, a major transformation in silicon nitride photonics is needed, where the ultra-low loss and wafer-scale CMOS foundry compatible processes of thin nitride structures and nonlinear properties of thick waveguide structures are maintained while adding the heterogeneous functionality of optical gain, high-speed modulation, electronics, and engineered thermal properties. At the same time, a uniform anneal-free waveguide fabrication process for both thin and thick structures is required. Heterogeneous and monolithic integration of thin and thick nitride photonics with materials that cannot withstand high annealing temperatures is inhibited by incompatibility with the high-temperature nitride growth and high-temperature post-oxide cladding annealing process used to achieve today’s low losses. Heterogeneous and monolithic integration material platforms of interest include silicon photonic circuits^30^, GaAs and InP semiconductor circuits^31,32^, and nonlinear materials such as lithium niobate^33^ and tantalum pentoxide (tantala)^34^ as well as materials for thermal engineering such as quartz substrates^35^. For example, efforts to limit the process temperature to under 400 °C can prevent crystallization in nonlinear tantala waveguides^34^ and enable processing waveguides directly on silicon electronics, silicon photonic circuits^30,36^, thin film lithium niobate^33^, and III–V semiconductors^31,32,37^. Further limiting processing temperatures to 250 °C enables a much broader class of heterogeneous and monolithic cointegration with organic electronics^38^, polymers like polyimide (Kapton)^39^, prepackaged electronics^40^, and substrates that are damaged under thermal stress like quartz^35,41^.

Therefore, heterogeneous and monolithic integration requires a uniform anneal-free silicon nitride fabrication process that can produce a wide range of nitride core thickness waveguides, of over an order of magnitude range, all while maintaining the loss and other planar and high-performance platform properties without additional process complexities such as stress mitigation and chemical mechanical polishing (CMP). State-of-the-art thin (<100 nm) waveguide silicon nitride photonics are essential to achieve the lowest losses that today reach 0.034 dB m^−1^ in the infrared^42,43^ and sub-dB m^−1^ losses in the visible^44^. These dilute mode ultra-low loss thin waveguides are required for precision applications, such as laser frequency stabilization and noise reduction, for example, integrated waveguide reference cavities yielding 36 Hz integral linewidth^45^ and stimulated Brillouin lasers (SBLs) with sub-100 mHz fundamental linewidth^21^. This level of performance is achieved by reducing the overlap of the optical mode with the etched nitride sidewalls and employing low-pressure chemical vapor deposited (LPCVD) silicon nitride waveguides patterned on top of a thermal silicon dioxide lower cladding and a tetraethyl orthosilicate-plasma enhanced chemical vapor deposited (TEOS-PECVD) upper cladding^42,43^. Yet, these processes require nitride growth temperatures as high as 850 °C^46^ and annealing temperatures of 1150 °C^1,42,47^. Recent efforts to reduce the process temperatures of these dilute waveguides employed an unannealed deuterated upper cladding oxide, however, still required 1050 °C annealing of the LPCVD nitride core in order to yield losses of 1 dB m^−1^^48^.

For high optical confinement thick nitride devices, the mainstay of nonlinear optical photonics, losses are determined primarily by sidewall scattering and nitride absorption. Thick core nitride waveguide designs utilize strong confinement to achieve efficient optical nonlinearities^15,49–51^, achieving losses as low as 0.4 dB m^−1^ and resonator Q as high as 67 million^52^. However, these typically require annealing temperatures of 1050 °C and structures for stress mitigation as well as CMP^53^. Research to reduce the processing temperature of thick nitride waveguides has focused on deuterated silicon nitride to lower losses in the nitride core only and has not addressed lowering the deuterated oxide cladding losses^54–59^. Therefore, these processes are not capable of realizing ultra-low loss and high resonator Q thin core (<100 nm) waveguides and devices. Examples of low-temperature thick nitride waveguides include 270 °C deuterated nitride with losses down to 22 dB m^−1^ and quality factors of 1.6 million for partially etched 920 nm thick waveguides^56,60^. More recently, 270 °C deuterated nitride yielded 6 dB m^−1^ loss and 5.3 million intrinsic Q in 850 nm-thick waveguides for 480 μm radius resonators and 11.9 dB m^−1^ loss and 2.9 million intrinsic Q for 150 μm radius resonators (see Supplementary Section S6)^58^. The first Si-rich deuterated thick nitride waveguides demonstrated losses of 150 dB m^−1^ and resonator intrinsic Q of 1.32 × 10^5^ with a 350 °C process^54,55,61^. Hydrogen-free low-temperature sputtering has also been employed, combined with 300 °C deposited upper cladding, to achieve 32 dB m^−1^ losses and 1.1 million intrinsic Q in 750 nm core waveguides^62^. After 400 °C annealing these achieved 5.4 dB m^−1^ loss and 6.2 million intrinsic Q. These low-temperature processes were used to demonstrate efficient optical nonlinearities, including Kerr microcombs^56,57^, octave-spanning supercontinuum generation^56^, and nonlinear frequency generation with optical parametric oscillation (OPO) thresholds of 13.5 mW^57^ and OPO threshold per unit resonator lengths down to 23.6 mW mm^−1^^58^. Si-rich deuterated nitrides have a high nonlinear index yielding efficient nonlinearities such as an OPO threshold as low as 10 mW and 31.8 mW mm^−1^ threshold per unit resonator length^61^. To date, there has not been a demonstration of anneal-free silicon nitride waveguide fabrication that lowers loss for both the nitride core and oxide cladding to enable an order of magnitude range of ultra-low loss thin and thick waveguides, with a maximum temperature of 250 °C for flexible heterogeneous and monolithic integration.

In this work, we report a significant advance in silicon nitride integrated photonics, achieving the lowest loss to date for an anneal-free silicon nitride waveguide. Additionally, using a maximum oxide and nitride temperature of 250 °C, we demonstrate the dual-use capability for ultra-low loss linear and nonlinear waveguides, using the exact same fabrication process for waveguides with an order of magnitude variation in thickness (80–800 nm). Stress mitigation and CMP are not needed either. We confirm the shifted absorption peaks of our 250 °C-grown deuterated Si_3_N_4_ by using Fourier transform infrared (FTIR) spectroscopy (Supplementary Section S2). The 250 °C maximum temperature is compatible with a wide range of materials, including organics^38,39^. We report 1.77 dB m^−1^ loss and a ~15 million intrinsic Q for a thin 80 nm core waveguide, over half an order of magnitude lower loss than previous low-temperature nitride processes^58,63^, and 85 times lower loss than deuterated Si-rich nitrides^54^. For thick 800 nm waveguides, we report comparable to record-low 8.66 dB m^−1^ loss and 4.03 million intrinsic Q, which is 39% higher than low temperature deposited thick nitride devices with similar area, as well as resonators that are 7.5 times smaller in area than the equivalent record-high Q low temperature fabricated device^58^. Our thick nitride devices measure 17 times lower loss^54^ and 30 times higher Q than low-temperature deuterated Si-rich nitrides^61^. To demonstrate the quality of our anneal-free fabrication process, we report record performance linear and nonlinear applications for both ultra-low loss thin and thick nitride waveguides. For thin waveguides, we demonstrate a ring resonator optical reference cavity that reduces laser frequency noise by over four orders of magnitude using a Pound–Drever–Hall (PDH) lock. We measure 20 Hz^2^ Hz^−1^ at a 10 kHz frequency offset from carrier and reduction in the integral linewidth to under 1 kHz, a factor of over 20 times reduction over the free running linewidth. This is the first demonstration of laser stabilization using an anneal-free, low-temperature waveguide reference cavity, to the best of our knowledge. This performance is only possible by realizing low loss and high Q for a 5.36 cm long cavity, which was 20× longer than the longest low-temperature processed waveguide reported to date^58^. This made for a thermorefractive noise (TRN) floor^45^ that was 10^3^ times less than that of a typical thick nitride resonator because of the larger modal area of thin waveguides. We also confirm the quality of our 800 nm-thick nitride waveguides and resonators with demonstrations of (1) Resonant optical parametric oscillation (OPO) and Kerr-comb formation and (2) non-resonant supercontinuum generation. Anomalous dispersion is measured as well as four-wave mixing parametric gain, with a near-lowest reported threshold of 16.7 mW for silicon nitride waveguides made with a low temperature process. We also measure over 2-octave supercontinuum generation from 650 nm to 2.7 μm, which is similar to the record for annealed LPCVD nitride^64^. We report an OPO threshold per unit resonator length of 15.2 mW mm^−1^, lower than reported for low-temperature deposited thick waveguides^58^ and twice as low as deuterated Si-rich thick nitrides (see Supplementary S11 Table TS7)^61,65^. Significantly, our thin waveguide losses are comparable with that of unannealed LPCVD nitride thin core waveguides of the same geometry (Supplementary Section S9). This dual-use capability of our anneal-free process for both thin and thick-core linear and nonlinear devices, with high-performance loss, demonstrates the versatility of this platform and its application to future heterogeneous and monolithic photonic integration.

**Fig. 1:**
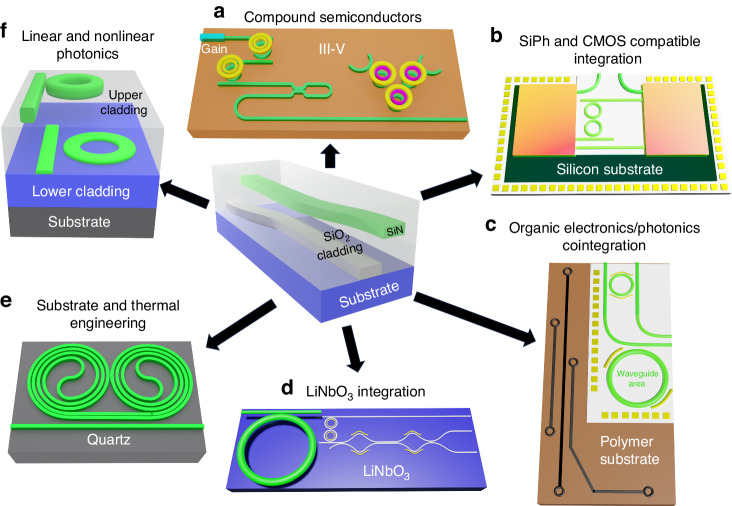
Examples of different applications of the anneal-free silicon nitride process. Cointegration with **a** compound semiconductors for high-performance lasers, **b** preprocessed silicon circuits and silicon photonics, **c** organic electronics/photonics, and **d** thin film lithium niobate. **e** Thermal and substrate engineering, such as with quartz substrates. **f** Homogenous integration of thick (>650 nm) and thin nitride core devices, each used for different applications

We illustrate examples of possible heterogeneous and monolithic integration (Fig. 1) enabled by our anneal-free process. These include the deposition of ultra-low loss waveguides on III–V semiconductors (Fig. 1a) for high-performance lasers and compound semiconductor photonic integrated circuits^66,67^, preprocessed electronic circuits, and silicon photonics^23,36^ (Fig. 1b), organic material based integrated circuits^68^ for cointegration with silicon nitride PICs and biophotonics^69^ (Fig. 1c), thin film lithium niobate^33^ (Fig. 1d), and materials like quartz for athermalization of resonators and reference cavities^35^ (Fig. 1e). Additionally, this process can be used to realize sophisticated multi-level silicon nitride photonic circuits^70^, homogeneously and monolithically integrated with other materials, to combine high-performance thin-waveguide components like spectrally-pure Brillouin lasers^17^ and thick waveguide nonlinear components including optical frequency combs^56,58^ (Fig. 1f).

## Results

### Anneal-free fabrication process and waveguide design

**Fig. 2:**
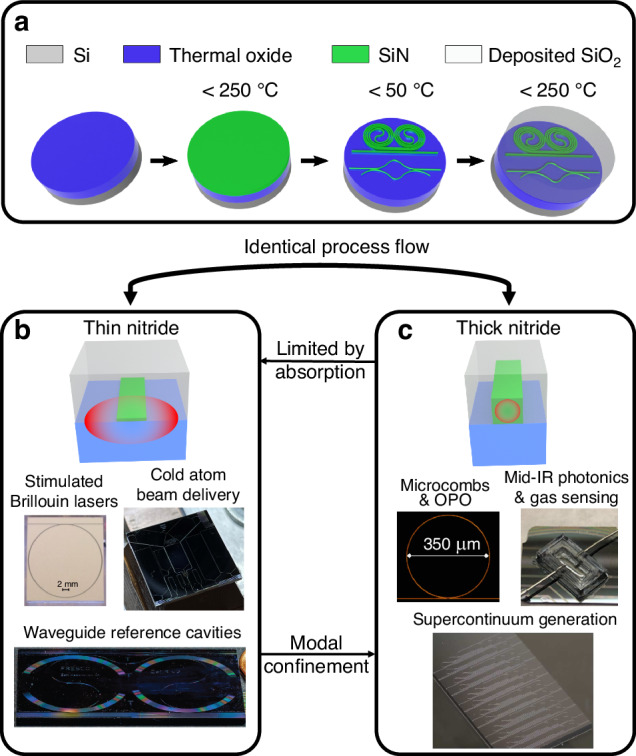
Anneal-free silicon nitride photonics integration process, thin and thick waveguides, and applications of each. **a** Anneal-free fabrication flow. The loss regimes vary between thin to thick waveguides in (**b**) and (**c**) respectively. **b** Example applications that require the performance of dilute mode thin nitride waveguides, with absorption-dominated losses, include stimulated Brillouin lasers^17^, coil resonator reference cavities^45^, and cold atom trap beam delivery^29,71^. **c** Examples of applications that require the characteristics of strongly confining thick nitride waveguides, with higher scattering dominated losses, include microcombs and optical parametric oscillators (OPO) (this work), mid-IR photonics and gas sensing^72^, and supercontinuum generation^56^

In this work, we demonstrate that the same process can be used to fabricate oxide-clad silicon nitride waveguides that have an order of magnitude variation in nitride core thickness, using the process flow shown in Fig. 2a, realizing the lowest anneal-free silicon nitride waveguide losses to date. This process is used for both thin and thick waveguides, and the wide range of core thickness enables device designs and functions that require different loss regimes and other optical characteristics, such as dispersion, to realize applications as shown in Fig. 2b, c. For example, thin ultra-low loss waveguides are required (Fig. 2b) for stimulated Brillouin lasers^17^, spiral resonator optical reference cavities^45^, and grating beam emitters for creating cold atoms^29,71^. Meanwhile, thick nitride waveguides (Fig. 2c) are required for OPO and microcombs such as in this work, supercontinuum generation^56^, and mid-IR photonics and gas sensing^72^. The process independence with respect to waveguide thickness, as well as anneal-free maximum temperatures of 250 °C, demonstrates the potential for co-integration of thin to thick nitride core devices and 3D monolithic and homogeneous integration^14,70^ as well as monolithic and heterogeneous integration on a variety of other material platforms.

The anneal-free process (Fig. 2a and described in further detail in the “Materials and methods” section) starts with a 1 mm-thick silicon wafer substrate with pre-processed 15 μm-thick thermal oxide lower cladding. A uniform silicon nitride layer (e.g. 80 or 800 nm) is then deposited using a deuterated silane precursor inductively coupled plasma–plasma enhanced chemical vapor deposition (ICP-PECVD) process at 250 °C. The nitride layer is patterned and etched at 50 °C using an inductively coupled plasma reactive ion etcher (ICP-RIE) etch. A final silicon dioxide cladding layer is deposited using the same deuterated silane precursor ICP-PECVD process at 250 °C. In the future, the lower cladding can also be deposited using our 250 °C process for co-integration with other materials and platforms. The different thicknesses of nitride devices correspond to variations in optical mode confinement from dilute modes for thin to strongly confining for thick. The thin waveguide losses are primarily dominated by absorption since nitride sidewall scattering is minimized due to low mode overlap with the core^43^, whereas the thick waveguide losses are dominated by sidewall scattering^14^. We can define “thin” waveguides as where the cladding absorption contributes 50% or more to the overall waveguide loss. The optical mode for thin nitride waveguides exists predominantly in the oxide cladding, therefore it is essential that the anneal-free fabrication process results in low losses for both the deposited nitride and oxide materials.

**Fig. 3:**
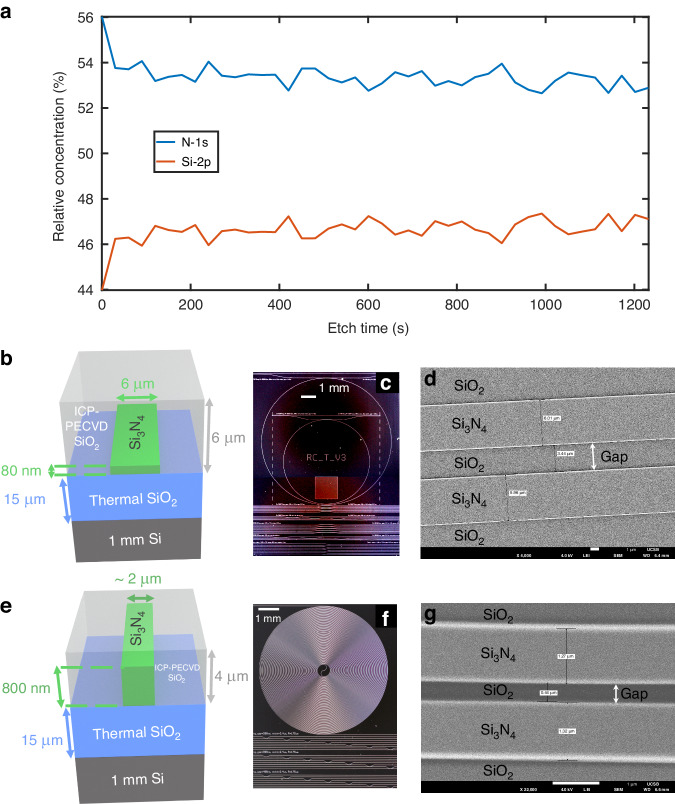
Material and device characterization and geometries for thin and thick nitride anneal-free devices. **a** SiN X-ray photoelectron spectroscopy (XPS) measurement showing a Si:N ratio of about 0.89:1. **b** Thin nitride waveguide geometry. **c** Thin nitride chip showing ring resonators, ring-bus coupling, and other test structures. **d** Top-down Scanning Electron Microscopy (SEM) image of the thin nitride waveguide with a width of 6 μm and gap of 3.5 μm on the mask. Measured gap is 3.44 μm, and waveguide widths are 6.01 and 5.96 μm, respectively. **e** Thick nitride waveguide geometry. **f** A thick nitride chip showing a bend loss spiral, ring-bus coupling, and other test structures. **g** Top-down SEM image of an 800 nm-thick nitride waveguide ring resonator, with a waveguide width of 1.4 μm and gap of 400 nm on the mask. The measured gap is 0.44 μm, and the measured waveguide widths are 1.32 and 1.27 μm, respectively. This confirms the high quality of our thick nitride waveguides

We characterize the absorption and composition of our anneal-free deposited nitride using FTIR^47,57^ (see Supplementary Section S2) and X-ray photoelectron spectroscopy (XPS)^73^. XPS measurements (Fig. 3a) show a 16 ± 1% nitrogen deficiency compared to commercial stoichiometric silicon nitride (see Supplementary Section S2 for more details), which can help increase the non-linear index and reduce thresholds for nonlinear effects^61^. We use a silicon nitride bus-coupled ring resonator configuration to access the anneal-free thin nitride losses and compare it to devices made with an unannealed LPCVD silicon nitride process (see Supplementary Section S9). The thin nitride waveguide design is a 6 μm wide, 80 nm-thick Si_3_N_4_ waveguide core with a 15 μm-thick thermal oxide SiO_2_ lower cladding layer and 5 μm-thick oxide upper cladding layer (Fig. 3b) for both the ring and bus waveguides. The ring radius is 8530.8 μm for the thin nitride chip, as shown in the example reference resonator photograph in Fig. 3c, and the ring-bus coupling gap is 3.45 μm as measured with scanning electron microscopy (SEM) prior to upper cladding deposition (Fig. 3d). The thin core waveguide is designed to support one quasi-transverse electric (TE) and one quasi-transverse magnetic (TM) mode irrespective of process parameter variations (see Supplementary Section S4). The waveguide design used for our anneal-free process is the same as that used in our standard fully annealed LPCVD nitride and TEOS-PECVD SiO_2_ process^74^. The thick nitride devices have an 800 nm-thick nitride core, a 15 μm-thick thermal oxide SiO_2_ lower cladding layer, and a 4 μm-thick oxide upper cladding layer (Fig. 3e). Design splits of the thick nitride devices include ring resonators with waveguide widths varying from 1.4 to 2.4 μm for both ring resonator and bus waveguides, ring radii varying from 165 to 177 μm, and ring-bus coupling gaps varying from 200 to 600 nm. Spiral waveguides were also fabricated with lengths of up to 35 cm, with a sample chip shown in the photograph in Fig. 3f. An example top-down SEM image of a ring resonator with a designed 2 μm waveguide width and 300 nm ring-to-bus waveguide gap is shown in Fig. 3g, indicating high-quality thick nitride deposition. Cross-sectional SEM images of the thick nitride core are also provided in Supplementary S2 Fig. S4.

### Thin nitride loss/Q and laser reference cavity application

**Fig. 4:**
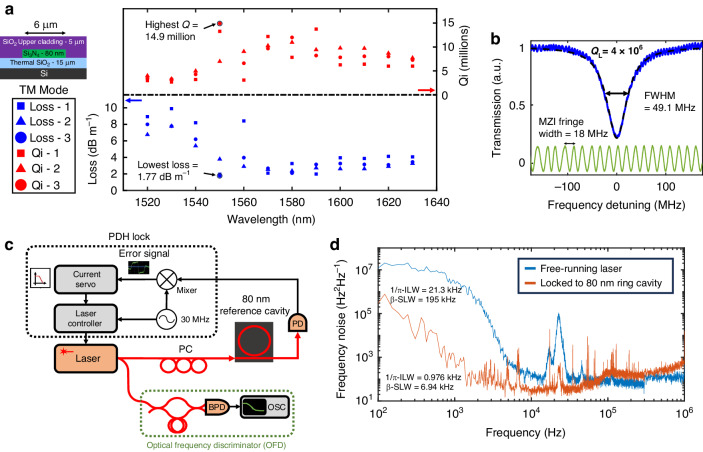
Thin nitride Q and loss measurements. **a** The loss and intrinsic Q variation of the transverse magnetic (TM) mode vs. wavelength for three different devices. **b** Q measurement of the TM mode resonance in device 3 at 1550 nm that yields the lowest loss and highest Q of 1.77 dB m^−1^ and 14.9 million, respectively. The loaded Q and full-width max (FWHM) are 4.0 million and 49.1 MHz, respectively. **c** The setup for the laser to resonator Pound–Drever–Hall (PDH) locking and frequency noise measurements with an optical frequency discriminator; PD photodetector, BPD balanced photodetector, PC polarization controller, OSC oscilloscope. **d** Frequency noise measurements at 1550 nm of the laser free-running vs. when PDH locked to the ring resonator cavity. The 1/*π*-integral linewidth (1/*π*-ILW) and β -separation linewidth (β-SLW) of the laser were reduced by factors of 22 and 28, respectively, upon locking. The frequency noise was as low as 20 Hz^2^ Hz^−1^ at 10 kHz frequency offset from the carrier, then

The waveguide losses and resonator Q are measured and calculated for the fundamental TM mode only, using a calibrated Mach Zehnder interferometer (MZI) technique^17,19,43^, and are described in further detail in the “Materials and methods” section. The group index used for this loss calculation was obtained from free spectral range (FSR) measurements of these devices^63^ and is 1.4642. For each thin-nitride resonator design, we characterize three different devices (Devices 1–3) and measure TM loss and Q for each device from 1520 to 1630 nm in steps of 10 nm (Fig. 4a). The minimum loss of 1.77 dB m^−1^ and maximum intrinsic Q of 14.9 million are measured at 1550 nm. The fabricated devices are over-coupled at wavelengths above 1540 nm, and the maximum Q corresponds to a 4.0 million loaded Q with 49.1 MHz FWHM resonance width (Fig. 4b). The median of the intrinsic Q and loss throughout the above wavelength ranges is 7.77 million and 3.26 dB m^−1^, respectively, while the average intrinsic Q and loss are 7.55 million and 4.31 dB m^−1^ respectively. Our lowest losses were over half an order of magnitude improvement compared to previous low-temperature deuterated devices^58^ and over 85 times better than existing deuterated Si-rich devices^61^.

Next, we report on a laser stabilization demonstration using PDH locking of a laser to the resonator device 3 at 1550 nm. The laser frequency noise is measured before and after locking using a calibrated MZI frequency noise discriminator^45^ (Fig. 4c, d). The measurement does not use vibration isolation, acoustic shielding, or temperature control of the resonator reference cavity. Hence low-frequency noise is dominated by environmental effects. The free-running laser 1/π-integral linewidth^75^ is 21.3 kHz, and the β-separation linewidth^75^ is 195 kHz. After PDH locking to the thin nitride resonator, the 1/*π*-integral linewidth is reduced to 0.976 kHz, a reduction factor of 22, and the β-separation integral linewidth is reduced to 6.94 kHz, a reduction factor of 28. Under PDH locking, the frequency noise is reduced by over 4 orders of magnitude at 0.67 and 1.4 kHz frequency offsets and as low as 20 Hz^2^ Hz^−1^ at 10 kHz frequency offset from the carrier. Further details of the setup and linewidth calculations can be found in Supplementary Section S8.

### Thick nitride loss/Q and OPO, FWM, and SHG nonlinear photonics applications

Nonlinear photonic waveguides with a wavelength-scale core thickness (~1 μm) offer the high optical confinement and waveguide dispersion needed for effective nonlinearities^15,50,52^. Our 800 nm-thick devices are fabricated using exactly the same process flow as described earlier for the thin nitrides, and the waveguide loss and Q measurements are performed as described in the previous section. We demonstrate that these anneal-free 800 nm nitride waveguides and resonators can achieve: (1) resonant OPO and Kerr-comb formation and (2) non-resonant supercontinuum generation. We simulate and measure the dispersion (see Supplementary S4 Figs. S6, S11 Fig. S17) and the losses of the different geometry variations in our devices, and based on these results, set the waveguide geometry to be 2 μm wide with a 300 nm bus to ring coupling gap, and the resonator radius to 175 μm.

**Fig. 5:**
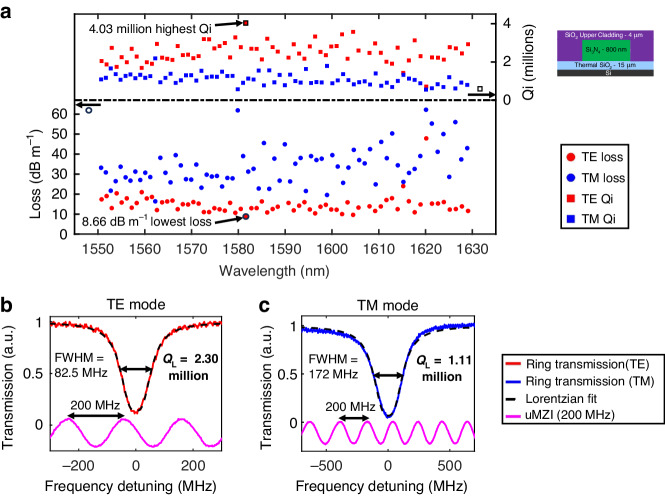
Thick nitride Q and loss measurements. **a** Loss and intrinsic Q variation vs. wavelength for the transverse electric (TE) and transverse magnetic (TM) modes from 1550 to 1630 nm wavelengths. **b** The TE mode resonance Q measurement at 1581 nm yielded the lowest loss of 8.66 dB m^−1^ and the highest intrinsic Q of 4.03 million. The loaded Q and full-width max (FWHM) are 2.3 million and 49.1 MHz, respectively. **c** The lowest loss TM mode resonance Q measurement at 1560 nm. All measurements shown here are for 175 μm radius ring resonators with 2 μm wide waveguides

The loss and Q values are measured for a wavelength range of 1550–1630 nm (Fig. 5a), using group indices of 2.025 and 2.053 for the TE and TM modes, respectively, which are obtained from measuring their respective FSRs. Example calibrated MZI resonance measurements for the lowest losses are shown in Fig. 5b, c for the TE and TM modes. These measurements yield losses as low as 8.66 and 16.4 dB m^−1^ and intrinsic Q as high as 4.03 million and 2.19 million, for the TE and TM modes, respectively. The loaded Q is measured to be 2.30 million and 1.11 with FWHMs of 82.5 and 172 MHz for the TE and TM modes, respectively. The median and average intrinsic Q, as well as loss for both polarization modes over the measurement wavelength range, are given in Table TS3 in Supplementary Section S6. Additional Q measurements for the TE mode around the wavelengths where the Q is maximum confirm that the same data points are not due to measurement error (see Supplementary S10 Fig. S13). In fact, these “outlier” wavelengths occur partially because higher-order modes do not interact with the fundamental modes^65^. We further perform measurements for the TE mode on three different devices with 165 μm radii with the same waveguide width and gap, which confirm that the loss and intrinsic Q measurements are repeatable from device to device (see Supplementary S10 Fig. S14). These losses are also confirmed in a longer spiral waveguide of length 0.35 m with Optical Backscatter Reflectometer measurements (Supplementary Section S10 Fig. S15), although the losses in the same are a few dB/m more due to waveguide defects that accumulate due to the long length^76^.

**Fig. 6:**
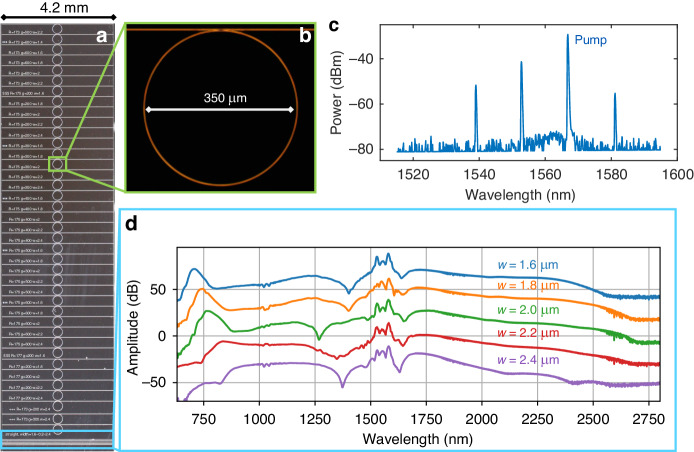
Nonlinear application demonstrations for anneal-free thick 800 nm waveguides and resonators. **a** Large field of view image of a thick nitride chip with a broad scan of ring resonator designs and straight waveguides. The green and blue highlighted regions correspond to devices tested in (**c**) and (**d**). **b** Dark-field optical micrograph of the ring resonator device used for Kerr-comb measurement. **c** Optical spectrum of the ring resonator output showing the onset of optical parametric oscillation. **d** Broadband supercontinuum spectra from the different width waveguides circled in light blue in (**a**)

For nonlinear applications of the thick nitride, we first demonstrate OPO and Kerr-comb formation in a 175 μm radius microring resonator. The resonator has a cross-sectional waveguide of dimensions 800 × 2000 nm (Fig. 6a, b), and we pump a resonance at 1566.7 nm, which has a measured *Q*_L_ ~ 1.6 million and *Q*_i_ ~ 2.0 million. Figure 6b shows an optical micrograph of one device. Figure 6c shows OPO at an on-chip pump power of 25 mW. As the pump power is increased, Turing pattern formation modulation-instability comb states^22^ are also observed (see supplementary section S11). We measure a threshold power, *P*_th_, for OPO of ~16.7 mW corresponding to an effective nonlinear index, *n*_2_ ~ 1.5 × 10^−19^ m^2^ W^−1^ (see “Materials and methods” section for more details), which is only slightly lower than typical measurements of *n*_2_ for stoichiometric nitride devices^77,78^. This corresponds to the lowest threshold power per unit length of 15.2 mW mm^−1^ for any low-temperature silicon nitride process, as well as for any previously reported Si-rich deuterated silicon nitride waveguide^61^ (see Table TS7 in Supplementary Section S11).

Next, we demonstrate broadband supercontinuum generation in 4 mm long, 800 nm-thick straight waveguides (Fig. 6d) with widths ranging from 1.6 to 2.4 μm. Figure 6d shows supercontinuum spectra measured by coupling light from a 1550 nm, 100 MHz repetition rate mode-locked laser with 100 fs pulse duration and on-chip pulse energies of ~200–400 pJ into the waveguides. The resulting supercontinuum emission covers two octaves, from ~650 to ~2.7 µm. CO_2_ absorption lines in the spectrum analyzer are evident at the long wave side of the spectrum. While the dispersion of these initial devices is not favorable for mid-infrared supercontinuum generation, we have measured absorption spectra of our deuterated nitride (Supplementary Section S2) and oxide layers^48^ and, in principle, our films should support waveguiding and supercontinuum generation out to 4 µm.

## Discussion

We report the lowest loss waveguides and highest Q integrated ring resonators, 1.77 dB m^−1^ loss and 15 million Q, fabricated with an anneal-free silicon nitride photonic low-temperature process with a maximum processing temperature of 250 °C for all steps. We demonstrate that this anneal-free process can be used for both thin and thick nitride waveguides, spanning a 10× thickness range, without requiring stress mitigation techniques or chemical mechanical polishing. Using the exact same process as record-low loss thin nitride waveguides, we achieve 8.66 dB m^−1^ loss and 4.03 million Q for 800 nm-thick nitride waveguides, the highest reported Q for a low-temperature processed resonator with equivalent device area (see Supplementary Section S6). We report both linear and nonlinear applications using thin and thick core resonators, demonstrating record performance for both types of applications and an anneal-free fabrication process. Laser noise reduction is demonstrated by PDH locking a laser to an ultra-low loss 80 nm thin nitride resonator employed as an optical reference cavity, achieving four orders of magnitude reduction in laser frequency noise. This was possible due to the more than order of magnitude larger modal area of thin nitrides compared to the thick and the long resonator length resulting in a TRN floor that was 10^3^ times smaller than a typical thick nitride resonator (see Supplementary Section S8). A high-Q 800 nm-thick nitride resonator is used to achieve resonant OPO with a 16.7 mW threshold corresponding to an OPO threshold per unit resonator length of 15.2 mW mm^−1^ and Kerr-comb formation, and over 2-octave non-resonant supercontinuum generation. The low 250 °C temperature and uniformity of this process across waveguide thickness and design, will enable a wide range of systems-on-chip applications and novel integration approaches. These include direct processing on organics, circuit cards, silicon photonic and III–V compound semiconductors, and lithium niobate, as well as enabling 3D integration stacking geometries that combine circuits with different nitride core thicknesses^14,70^.

The thin and thick nitride devices cover two different loss regimes, the thin dominated by absorption loss of the cladding material, and the thick by scattering loss and core absorption (Fig. 2b, c). We confirm this for our thin nitride devices at 1550 nm by measuring the thermal bistability for different on-chip powers giving us an absorption loss fraction of 59% corresponding to an absorption limited loss of 1 dB m^−1^ (Supplementary Section S7), comparable with 90 nm annealed LPCVD nitride cores with a deuterated oxide cladding^48^. We see that this satisfies the >50% cladding absorption definition of thin waveguides that we introduce in the section “Anneal-free fabrication process and waveguide design”. Previously reported work on thick core low-temperature nitrides using deuterated processes^56,58,61^ as well as sputtering^62^ did not demonstrate low absorption loss for their upper claddings and hence ultra-low loss thin nitride devices were not achieved. We note that we could reduce the nitride core thickness further to achieve losses closer to the cladding absorption limit; however, this would come at the expense of increased critical bend radius and device area. The absorption losses in our thin nitride devices are thought to be partially from the unannealed thermal oxide lower cladding, which can be further improved by depositing deuterated SiO_2_ for the lower cladding, a subject of future work. The small amount of hydrogen present in the deuterated silane precursor also increases the absorption loss, as evidenced by the increase in waveguide loss towards 1520 nm (Fig. 4a), which is near the 1st overtone of the SiN–H bond absorption. Towards 1630 nm, the loss increase is most likely due to overtones of the SiO–D bond in the upper cladding^48^. We additionally see that the thin losses are comparable to devices of the same geometry made with unannealed LPCVD nitride (Supplementary Section S9), confirming that our losses are competitive with respect to process temperature. The more tightly confined modes in the 800 nm-thick devices have higher sidewall scattering losses than their thin nitride counterparts and could be improved by using a hard mask with smaller grain size, such as those made with atomic layer deposition^79,80^ or RF sputtering^81^. The losses we achieve can be interpreted to be better than previous works^54,56,61^ that used ICP-PECVD grown nitride as our waveguide cores are smoother or closer to an ideal shape (Fig. 3g, Supplementary Fig. S4). The TM mode loss for the thick nitride is very different compared to the loss for the TE mode, as the top surface roughness of the nitride core is much lower than the etched sidewall roughness, and the two modes are significantly different in shape (see Supplementary Section S4). It should also be noted that our highest intrinsic Q thick nitride resonances exhibit resonance splitting (see Supplementary Section S6), which is believed to be due to the scattering loss fraction being higher at those resonance wavelengths^43^. In this process, we utilized ICP-PECVD with deuterated silane and nitrogen precursors for silicon nitride deposition, avoiding ammonia due to the concentrated inductively coupled plasma (ICP) induced dissociation of N_2_ that cannot be achieved with conventional parallel plate PECVD^82^, and eliminated hydrogen absorption losses^47,56,58,61^. Alternative low-temperature processes to ours include sputtering and conventional plasma-enhanced chemical vapor deposition (PECVD)^83,84^, but both suffer from high particle count-related scattering losses, and conventional PECVD-grown silicon nitride suffers from high hydrogen-related absorption losses due to using ammonia and silane precursors^84,85^. Our nitride is slightly silicon-rich, as seen in XPS measurements, but both the linear and nonlinear refractive indices (see Supplementary section S3) are closer to that measured for stoichiometric silicon nitride^84^. This behavior is typical for ICP-PECVD-grown silicon nitride^84,86^. Additionally, the Si-rich nature of our current nitride causes high absorption losses at visible wavelengths (see Supplementary Section S2); however, this could be rectified by introducing more N_2_ during nitride deposition.

**Fig. 7:**
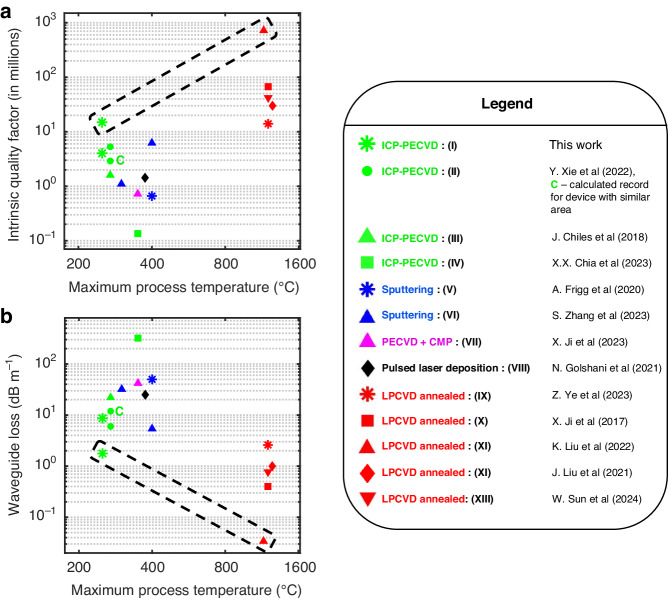
Q and loss vs. temperature near the C-band for different published works based on their silicon nitride growth methods and processing compared to this work. Our lowest losses (thin nitride) are near an “optimum”, denoted by the oval, between low loss and temperature, the current record low loss also being a thin nitride device^42^. Our thick nitride structures have double the Q of the current record calculated for low-temperature fabricated devices with similar areas^58^ as marked with the C, and very similar loss overall to the absolute record while having an area 7.5 times smaller. The different works compared include inductively coupled plasma-plasma enhanced chemical vapor deposition (ICP-PECVD) processes using deuterated silane precursors like (I) This work, (II) Y. Xie et al. ^58^, (III) J. Chiles et al. ^56^, and (IV) X.X. Chia et al. ^61^—which also uses Si-rich SiN; Sputtering such as (V) A. Frigg et al. ^83^, (VI) S. Zhang et al. ^62^; plasma enhanced chemical vapor deposition (PECVD) in conjunction with chemical–mechanical polishing (CMP) (VII) X. Ji et al. ^90^; pulsed laser deposition—(VIII) N. Golshani et al. ^91^; And low-pressure chemical vapor deposition (LPCVD) together with annealing, such as (IX) Z. Ye et al. ^92^, (X) X. Ji et al. ^52^, (XI) K. Liu et al. ^42^, (XII) J. Liu et al. ^76^— which uses a Damascene process too, and (XIII) W. Sun et al. ^93^

A summary of published losses and intrinsic Q near the C-band of ring resonators made with different processes as a function of maximum processing temperature and their nitride processing methods is given in Fig. 7, and compared to this work. Our reported lowest losses fall in an “optimum” region between loss and process temperature. It should be noted also that the record low-loss thick nitride devices had a width of 10 µm^52^. The full details of all the previous works compared in Fig. 7 can be found in Supplementary Section S12. Our anneal-free process, with a maximum processing temperature of 250 °C, and uniformity for core thickness spanning an order of magnitude, is fully CMOS-compatible and will pave the way to monolithic and heterogeneous integration of ultra-low loss silicon nitride photonics with material systems not possible before such as III–V semiconductors^31,37^, lithium niobate^33^, preprocessed silicon circuits and photonics^40^, and organic electronic materials^39^. This will allow for applications in metrology^9^, navigation^8^, telecommunications^10^, quantum information sciences^2–4^, and consumer electronics where organic electronics is widely used^87^. This process could also be used to monolithically and homogeneously integrate both thin low-confinement and thick high-confinement silicon nitride waveguides, enabling 3D integration with optimized device footprint and linear and nonlinear performance. In the future, the temperature of our process has the potential to be modified for as low as 50 °C using further process development on our ICP-PECVD tool^88^ (which supports 50 °C processes), enabling the monolithic integration of ultra-low loss photonic integrated circuits on most organic electronic materials.

## Materials and methods

### Fabrication process

The thick and thin SiN core and SiO_2_ upper cladding depositions are performed using an Unaxis VLR ICP-PECVD tool with the same processes used for all core thicknesses and devices. Further details on the nitride deposition and oxide deposition processes can be found in Supplementary Section S1. Before any deposition on a device wafer, we run a deposition on a test 100 mm silicon wafer and measure the particle counts with a KLA/Tencor Surfscan, as well as the film thickness and refractive index with a Woollam ellipsometer. The deposition on the device wafer is performed only if the particle counts increase by <300. The fabrication starts with the 250 °C silicon nitride deposition on Si wafers with 15 μm of thermal oxide, with the thick nitride depositions merely being done for longer than the thin nitride deposition, in a single step. After the nitride deposition step the thick nitride wafers only, get 40 nm of Ruthenium DC sputtered in an AJA ATC 2200-V sputter system. Both the thick and thin nitride wafers are then patterned in a 248 nm ASML PAS 5500/300 DUV stepper, using the same lithography parameters. The thin nitride is then etched in a Panasonic E640 ICP-RIE using a CF_4_/CHF_3_/O_2_ chemistry, after which it is ashed in an O_2_ plasma in a Panasonic E626I ICP tool to remove etch byproducts. Any remaining photoresist is stripped by sonicating in a hot N-methyl-2-pyrrolidone (NMP) solution and rinsing in isopropanol. We additionally perform a standard piranha clean at 100 °C followed by a base piranha (5:1:2 solution of H_2_O:NH_4_OH:H_2_O_2_) clean at 70 °C, both in freshly prepared solutions, making the thin nitride wafers ready for upper cladding deposition. For the thick nitride fabrication, the Ru on the thick nitride is etched in a Panasonic E640 ICP-RIE, too, to create a hard mask, using a Cl_2_/O_2_ chemistry. The thick nitride wafer is then stripped of photoresist the same way as the thin one, using a hot NMP solution and isopropanol. It is then etched in a Plasma-Therm 770 SLR, an ICP-RIE using CF_4_ only, after which the same O_2_ plasma ashing as the thin nitrides is done. Any remaining Ru is stripped in a wet etch using Transene RU-44 etchant, and then the same piranha cleans done for the thin nitrides are performed. The requisite amount of ICP-PECVD SiO_2_ upper cladding is then deposited at 250 °C on both the thin and thick nitride wafers. The flow diagrams of these fabrication processes can be found in Supplementary Section S5.

### Quality factor measurements and calculation

The loaded quality factors of the ring resonators are measured using three different calibrated unbalanced fiber MZIs with MZI fringe widths of 5.87, 18, and 200 MHz. We have seen in our previous works that Q values measured with this method match well with cavity ring-down measurements^89^. Two Newport Velocity TLB-6700 tunable lasers are used, one with a tuning range of 1520–1570 nm, and another one with a tuning range from 1550 to 1630 nm. These lasers are tuned in wavelength with piezo actuators, by applying a ramp signal to the same. A polarization controller is present before the input to the thin nitride devices, which is edge-coupled to a single-mode cleaved fiber, while there is a polarization beam splitter present before the input to the thick nitride devices. The full setup for the thin nitride Q/loss measurements is shown in Supplementary Section S6 Fig. S9. Loaded and intrinsic quality factors are extracted by fitting the resonance transmission to a Lorentzian (thin nitride) or coupled-Lorentzian (thick nitride) curves. Coupling and loss parameters are determined by measuring the ring-to-bus couplings on independent ring-bus coupling structures as well as simulating the same^35^. Additional details can be found in Supplementary Section S6, and plots of all resonance measurements in part 2 of the supplementary—Resonance Measurement Summary section.

### Threshold power for optical parametric oscillation

We determine the effective nonlinear index for our deuterated nitride by measuring the threshold power for OPO, *P*_th_, according to the following^15^:

$${n}_{2}=\frac{{\pi }{n}{v}_{0}\,{A}_{{{eff}}}}{8\,{P}_{{{th}}}\,{v}_{{{FSR}}}\,{Q}_{{i}}^{2}}\frac{{(1+K)}^{3}}{K}$$

where *n* is the effective refractive index, A_*eff*_ is the effective mode area, ν_*FSR*_ = 133.5 GHz is the resonator-free spectral range, v_*o*_ is the pump frequency, *Q*_*i*_ is the resonator intrinsic *Q*, and *K* is a resonator coupling constant *K* = *Q*_*i*_/*Q*_c_, where *Q*_*c*_ is the resonator coupling *Q*. We extract values of *Q*_*i*_ and *Q*_*c*_ through the Lorentzian curve fitting method described above. We then use the software Lumerical MODE to calculate A_*eff*_ and *n* as a function of wavelength (in this case, 1.35 µm^2^ and 1.85, respectively). Based on our analysis, we determine *n*_2_ ~ 1.5 ± 0.2 × 10^−19^ m^2^ W^−1^. Measurement uncertainty is propagated from the measurement resolution of the threshold power and the one standard deviation error of the curve fitting parameters, which determine *Q* values.

### Supplementary information

Supplementary information for Anneal-free ultra-low loss silicon nitride integrated photonics

## Data Availability

The data that support the plots within the paper and other findings of this study are available from the corresponding author upon reasonable request.
