# Supplementary material for: Anneal-free ultra-low loss silicon nitride integrated photonics
Source: Light Sci Appl. 2024 Jul 8;13:156. doi: 10.1038/s41377-024-01503-4 (PMC11231177; doi:10.1038/s41377-024-01503-4)
Supplement: Supplementary file 1 — Supplementary information for Anneal-free ultra-low loss silicon nitride integrated photonics [file 41377_2024_1503_MOESM1_ESM.docx]

**Supplementary information for Anneal-free ultra-low loss silicon nitride integrated photonics**

**Debapam Bose^1^, Mark W. Harrington^1^, Kaikai Liu^1^, Andrei Isichenko^1^, Jiawei Wang^1^, Nitesh Chauhan^1^, Zachary L. Newman^2^, and Daniel J. Blumenthal^1*^**

*^1^Department of Electrical and Computer Engineering, University of California Santa Barbara, Santa Barbara, CA 93106, USA*

*^2^Octave Photonics, Louisville, CO 80027, USA*

** danb@ucsb.edu*

**Table of Contents**

**Part 1 :**

**Section S1 : ICP-PECVD processes and development.**

**Section S2 : Film material Characterization.**

**Section S3 : Refractive indices of materials.**

**Section S4 : Waveguide mode and dispersion simulations.**

**Section S5 : Fabrication process flow.**

**Section S6 : Quality factor measurement and loss extraction/calculation.**

**Section S7 : Absorption loss estimation**

**Section S8 : Thermorefractive Noise (TRN) Floor Estimation, PDH locking and frequency noise measurements**

**Section S9 : Thin nitride loss comparison with LPCVD nitride.**

**Section S10 : Additional Q/loss measurements of thick nitride devices**

**Section S11 : Calculations and additional non-linear application measurements of thick nitride devices**

**Section S12 : Comparison table of losses/Qs between different processes

Part 2 :**

**Supplementary : Resonance Measurement Summary**

**P1.**

**S1. ICP-PECVD processes and development**

The 250 °C nitride deposition step uses deuterated silane, nitrogen, and argon respectively, at a plasma RF power of 800 W, and at a pressure of 5 mT with no substrate bias. The deuterated silane used is measured to have an isotropic purity of 99 %. Before running any actual device wafer, a seasoning process is run with a non-device wafer to coat the chamber. Further, before the nitride deposition step on a device wafer, an Ar preclean is run with said device wafer in the chamber. Particle counts added to wafers after nitride deposition are measured over a 100 mm wafer for sizes between 160 nm to 1.6 μm to be less than 300 consistently using a KLA/Tencor Surfscan. The nitride film etches at a rate of 7.1 nm min^-1^ in a Transene UN2817 buffered HF solution, and the deposition rate of the film in the ICP-PECVD tool is measured to be 42 nm min^-1^ using an ellipsometer. For a 336 nm nitride film on a 100 mm silicon wafer, the compressive stress is measured to be 666 MPa using a Tencor Flexus FLX-2320 film stress measurement tool.

The 250 °C oxide deposition step is very similar to the work by Jin et al^1^ and uses deuterated silane and oxygen respectively at a plasma RF power of 800 W, and at a pressure of 5 mT with 15 W of substrate bias. Seasoning and argon preclean steps are also done before oxide deposition on actual wafers, as well as measurement of particle counts and stress etc. The process is regularly characterized by the UCSB cleanroom also^2^.

**S2. Film Material Characterization**

FTIR measurements are carried out in transmission mode at normal incidence, in a purged chamber, using a Bruker Vertex 70 FT-IR Spectrometer. The transmission data is normalized against a Si wafer, and Fabry-Pérot oscillations are removed using a thin film transfer matrix model to get the data as shown in Fig. S1. The shifted SiN-D absorption peak of our silicon nitride film at 4 μm is due to deuteration instead of the absorption peak seen near 3.04 μm for hydrogen-based SiN-H deposition^3^, confirming that the 1st overtone occurs near 2 μm instead of near 1.52 μm for SiN-H.


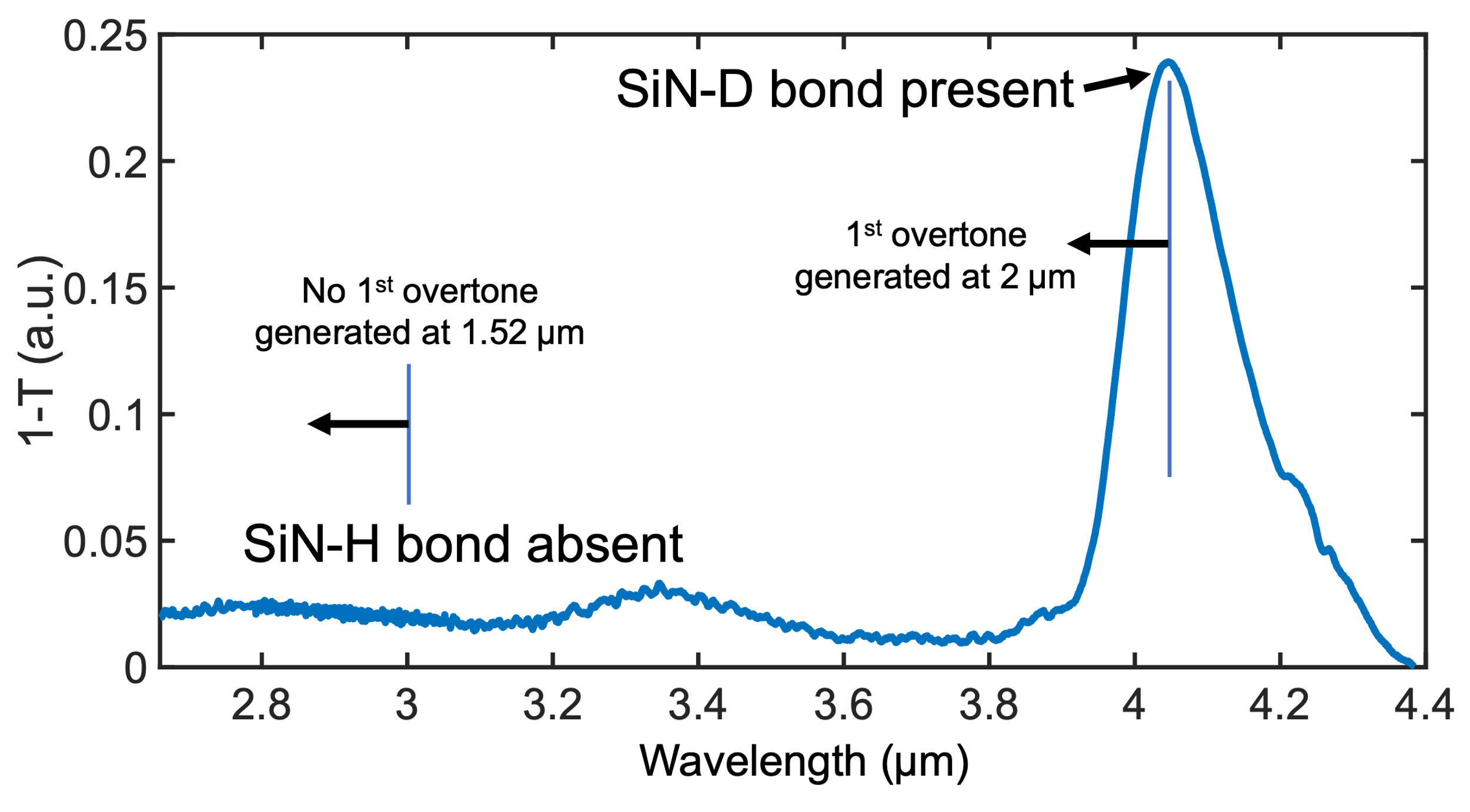


**Fig. S1 Deuterated SiN Fourier Transform Infrared Spectroscopy (FTIR) measurement.** There is no peak near 3.04 μm, and a peak at 4 μm instead due to deuteration. This causes the first overtone of the same peak to be near 2 μm instead of at 1.52 μm.

XPS measurements are done in a partially evacuated chamber using a Kratos Axis Ultra X-ray Photoelectron Spectroscopy (XPS) system. For our ICP-PEVD nitride the data was recorded for about 1200s, in which about 300 nm of the ICP-PECVD SiN was etched into. There is about 1.5 % of Oxygen that was also detected, not plotted in Fig. 3a, which is believed to be due to diffusion into the nitride^4^. The XPS tool cannot detect Argon which is one of the process gases used for the nitride growth, as it is a noble gas, or deuterium. The Si:N ratio thus, from fitting, had already been calibrated to commercially grown stoichiometric LPCVD nitride, the XPS measurement of which is in Fig. S2 below. This LPCVD nitride XPS survey was taken 114 nm from the surface, into a film with a total thickness of 175 nm, after etching with the XPS tool in steps.


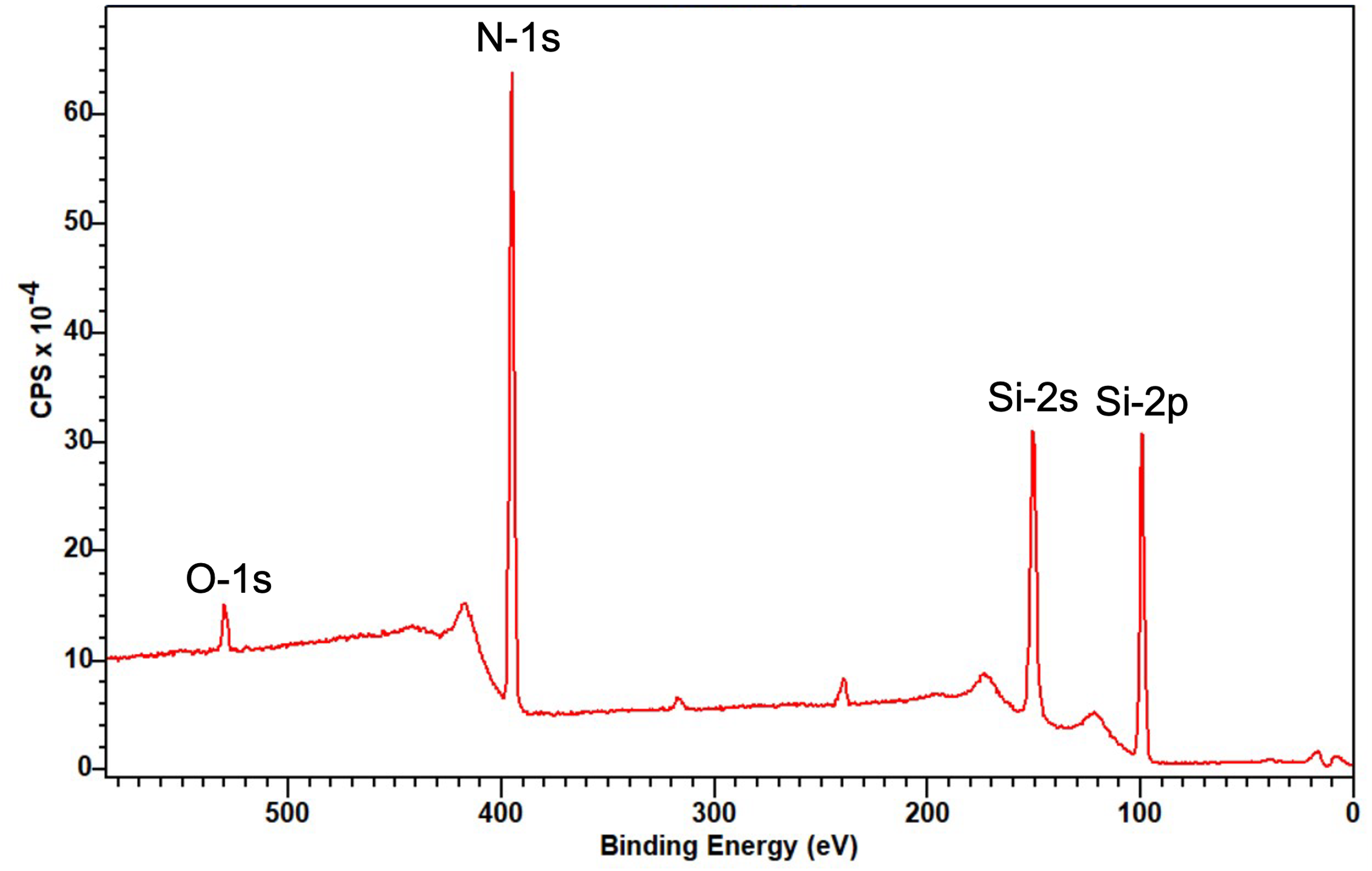


**Fig. S2 Stoichiometric LPCVD SiN X-Ray Photoelectron Spectroscopy (XPS) measurement.** for a commercially grown stoichiometric LPCVD nitride film. The XPS survey is at 114 nm deep into the LPCVD nitride film after etching with the XPS tool, where the Si:N ratio had stabilised.The main peaks seen are for Si and N species.

The higher than stoichiometric Si content we see in our ICP-PECVD Si_x_N_y_ XPS measurement is corroborated by loss measurements of 900 nm wide x 120 nm thick waveguides that support both 780 nm and 1550 nm modes, in which the loss at 780 nm is of the order of dB mm^-1^, while the loss at 1550 nm is on the order of only 10 dB m^-1^, as silicon has very high absorption at visible wavelengths as we see in Fig. S3 below. The loss at 1550 nm is measured using Optical Backscatter Reflectometry (OBR) on a 0.75 m long spiral waveguide, while the loss at 780 nm is obtained from the cutback method on waveguides of lengths on the order of a cm as meter length waveguides have too high an absorption at 780 nm to measure loss accurately. To reliably get ultra low loss at visible wavelengths with our process, the nitride deposition recipe used must be modified.


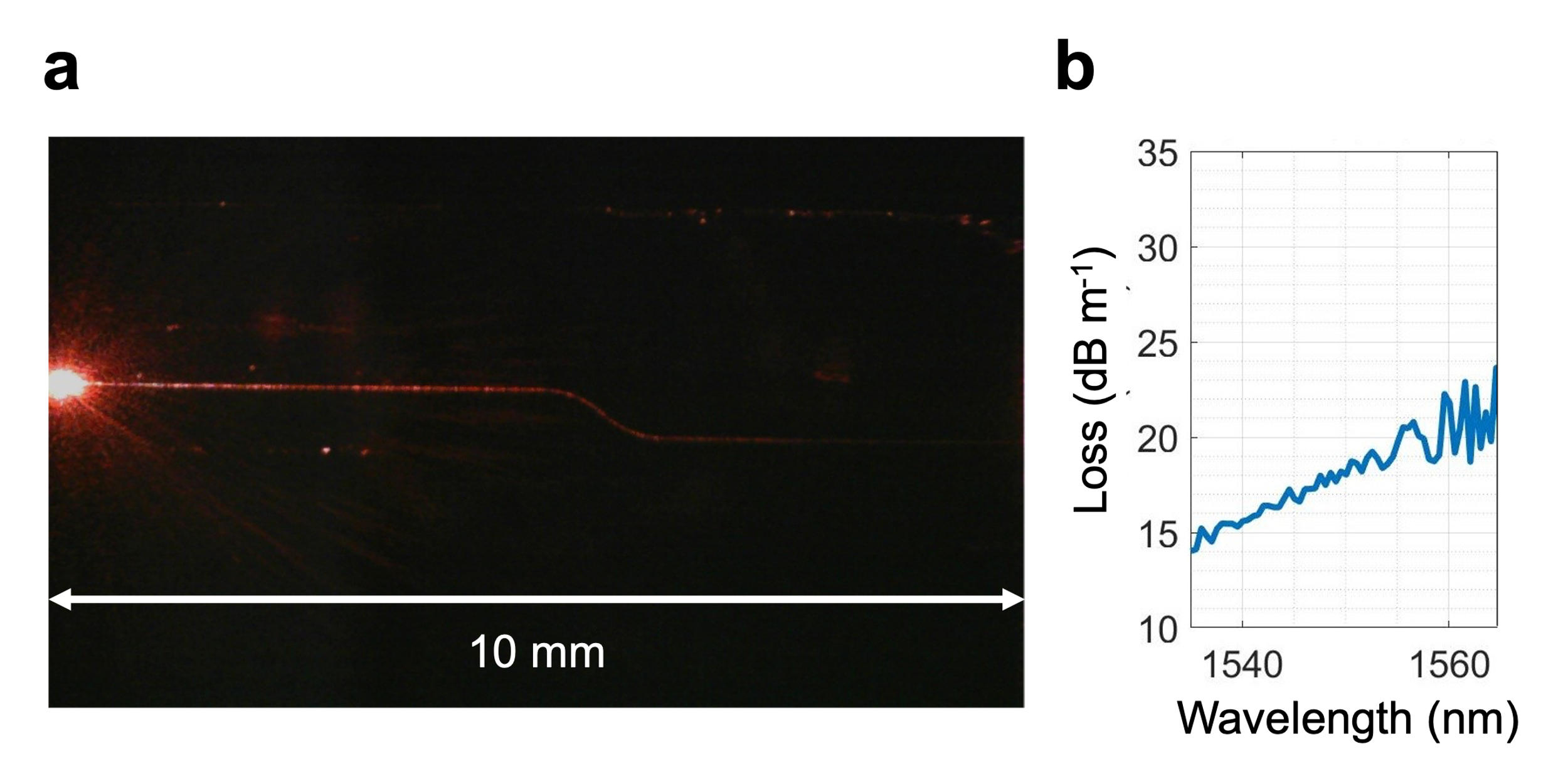


**Fig. S3 Visible vs near-IR comparison of waveguides.** We use a 900 x 120 nm waveguide geometry to compare the loss of our process between visible and near-IR wavelengths. **a** 780 nm light is seen getting absorbed on lengths on the order of a cm **b** The loss in near-IR is on the order of 10s of dB m^-1^.

We also take cross-sectional SEM measurements of our 800nm thick nitride waveguides confirming the dimensions and quality of these waveguides as in Fig. S4 below.


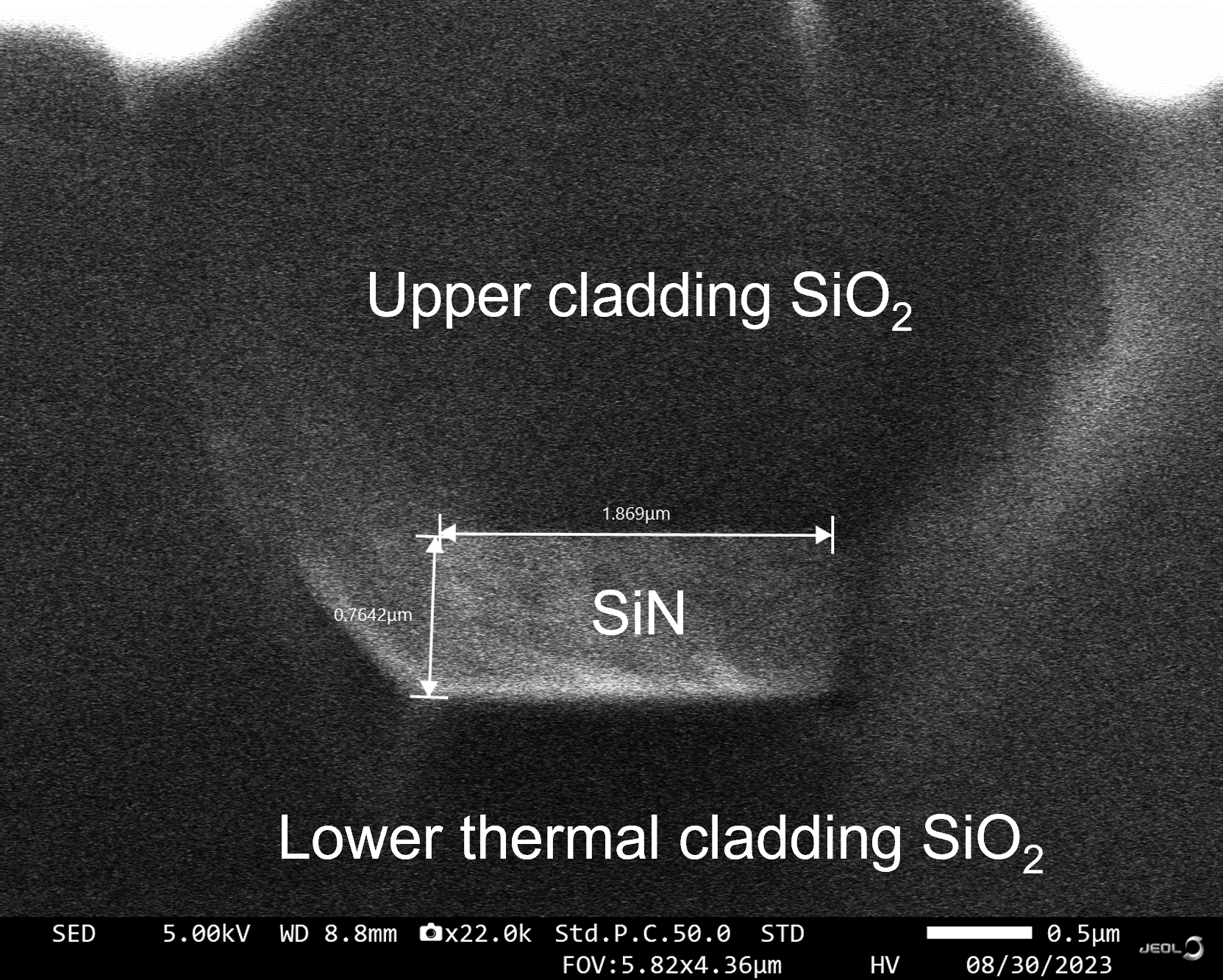


**Fig. S4 Cross-sectional SEM of our 800 nm thick nitride waveguides.** Cross-sectional SEM of a 800 nm thick waveguide with 2 μm wide width on mask.

**S3. Refractive indices of materials**

The thin and thick nitride devices are fabricated more than 1.5 years between each other in a university cleanroom, and hence the indices of the deposited materials are slightly different even if using the same recipe, as given below in tables TS1 and TS2. All measurements are from a Woollam Ellipsometer. While the nitride recipe was being developed, XPS measurements were not taken; however a refractive index of 1.95 was targeted as that is similar to stoichiometric LPCVD nitride we have used in previous works^5^.

**Table TS1. Refractive indices of different materials for thin ICP-PECVD nitride core devices at 1550 nm**

| **Material** | **Si_3_N_4_** | **SiO_2_ lower cladding** | **Upper cladding ICP-PECVD SiO_2_** |
| --- | --- | --- | --- |
| **n** | 1.95 | 1.445 | 1.456 |

**Table TS2. Refractive indices of different materials for thick ICP-PECVD nitride core devices at 1550 nm**

| **Material** | **Si_3_N_4_** | **SiO_2_ lower cladding** | **Upper cladding  ICP-PECVD SiO_2_** |
| --- | --- | --- | --- |
| **n** | 1.963 | 1.445 | 1.459 |

**Section S4 : Waveguide mode and dispersion simulations**

Figure S5 below shows mode simulations for the TM mode for the 80 nm x 6 μm thin and both TE and TM modes for the 800 nm x 2 μm thick nitride core devices respectively, from Lumerical MODE solver.


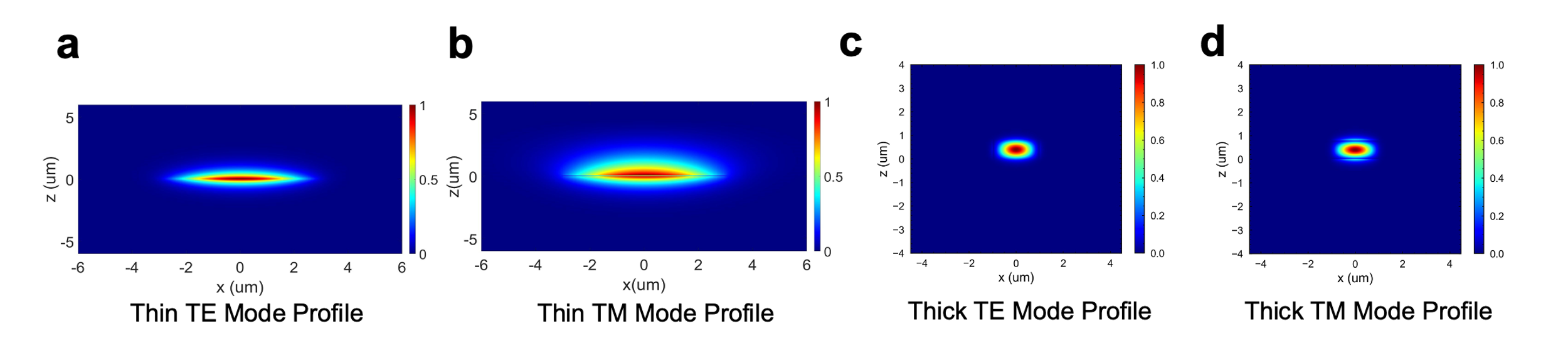


**Fig. S5 Mode simulations.** for **a** 80 nm Transverse electric (TE) Mode **b** 80 nm Transverse magnetic (TM) Mode **c** 800 nm TE Mode **d** 800 nm TM Mode. The 800 nm nitride

Below are the effective areas of different modes for the thin and thick nitrides. The order of magnitude larger modal volume of the thin nitride mode makes it ideal for use as a reference cavity, while the smaller thick nitride modes are better for exploiting the Kerr effect.

**Table TS3. Modal area of different modes**

| **Mode** | TM | TE | TM |
| --- | --- | --- | --- |
| **Core Thickness** | 80 nm | 800 nm | 800 nm |
| **Modal Area** | 27 μm^2^ | 1.35 μm^2^ | 1.66 μm^2^ |

Using index data for our nitride and oxide films measured via ellipsometry, Lumerical MODE solver was used to calculate dispersion curves for our 800 nm thick waveguides for a range of widths from 1.5 μm to 2.5 μm, in steps of 0.5 μm for 800 nm of nitride. Figure S6 shows dispersion data for our thick nitride structures which exhibit anomalous dispersion near 1550 nm (red dashed line).


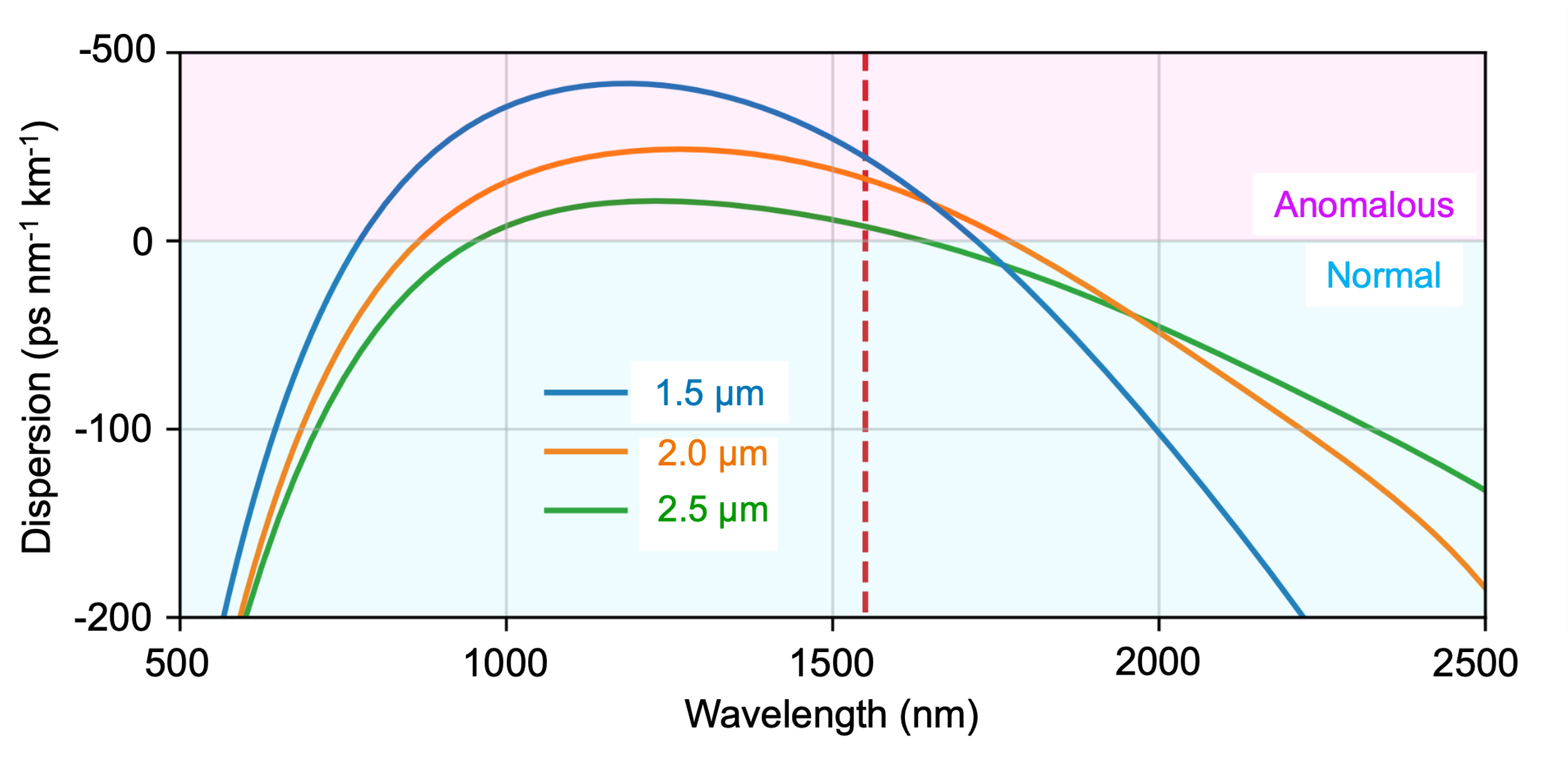


**Fig. S6 Waveguide dispersion simulations for different waveguide widths**

**Section S5 : Fabrication process flow**

Figure S7 below shows our complete fabrication process flow for our thin nitrides. The etching process we use for the same is similar to what we use in our previous works with LPCVD nitride^5^, and should only be used if etching less than 200 nm of nitride due to the selectivity of the deep UV Photoresist mask to the nitride we have.


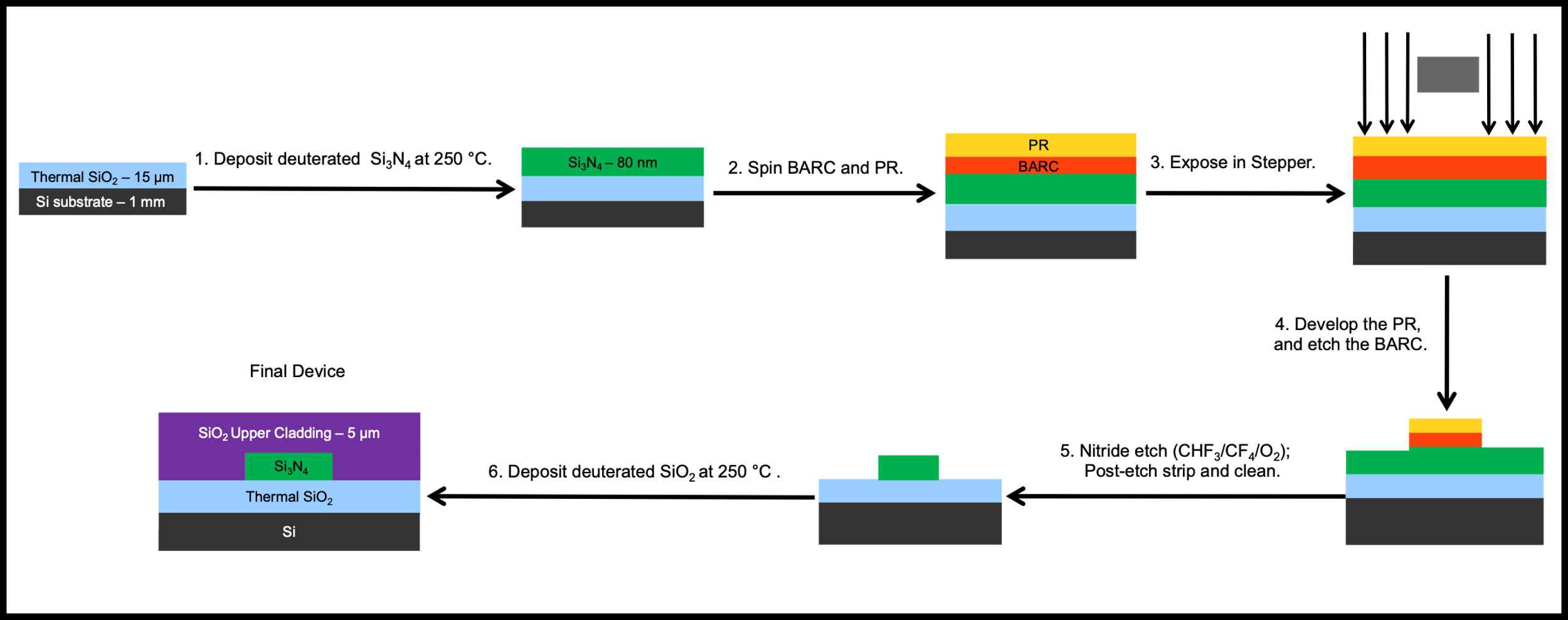


**Fig. S7 Detailed fabrication process flow for thin nitride devices.** PR = Photoresist, BARC = Bottom anti-reflective coating.

Figure S8 below shows our complete fabrication process flow for the thick nitrides.


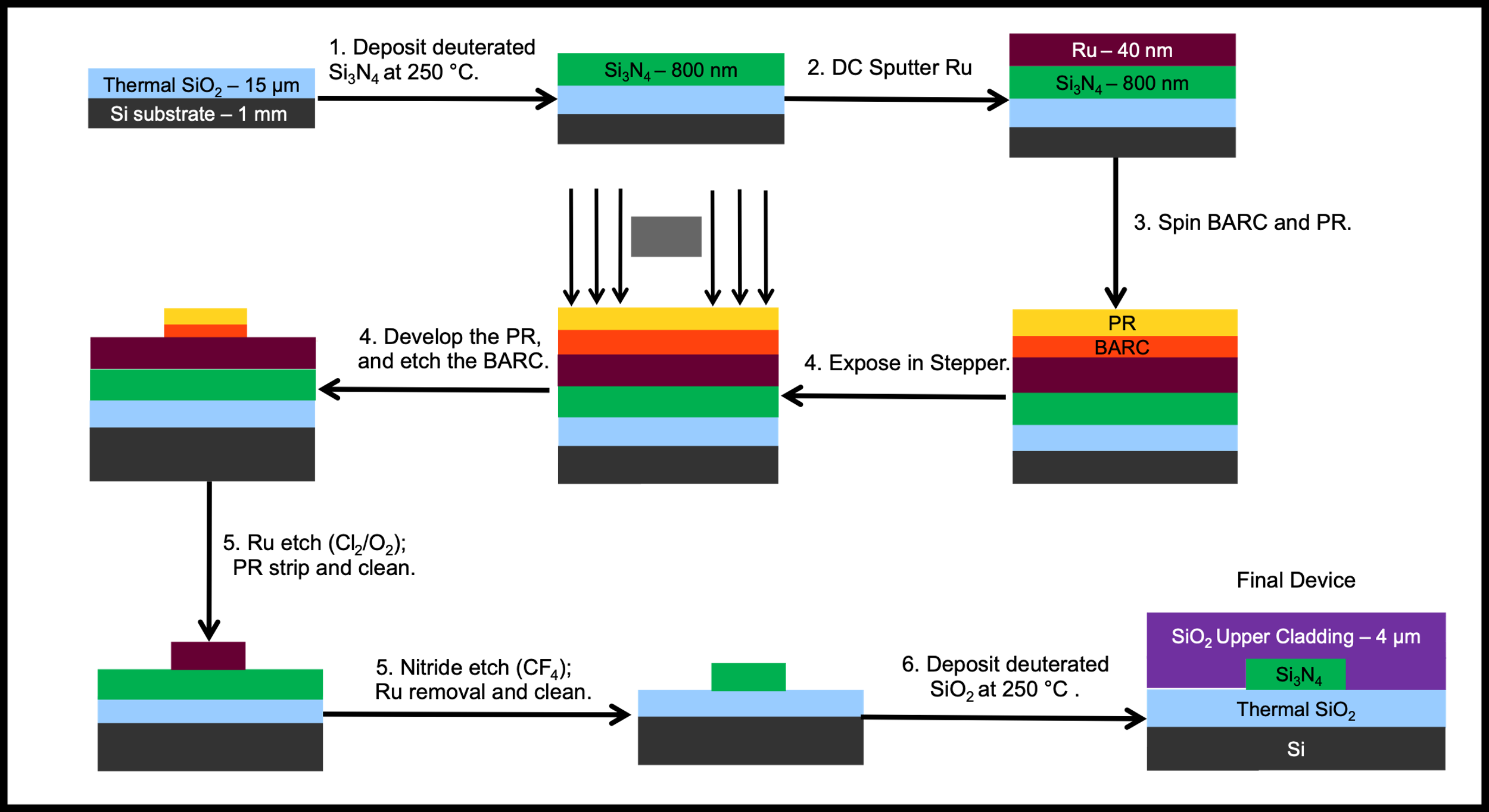


**Fig. S8 Detailed fabrication process flow for thick nitride devices**

**S6. Quality factor measurement and loss extraction/calculation**

The calibrated Q setup used to measure the Quality factors and loss for the thin nitrides is given Fig. S9 below. The setup for the thick nitrides is exactly the same, except it uses a polarization beam splitter before the Device-Under-Test (DUT).


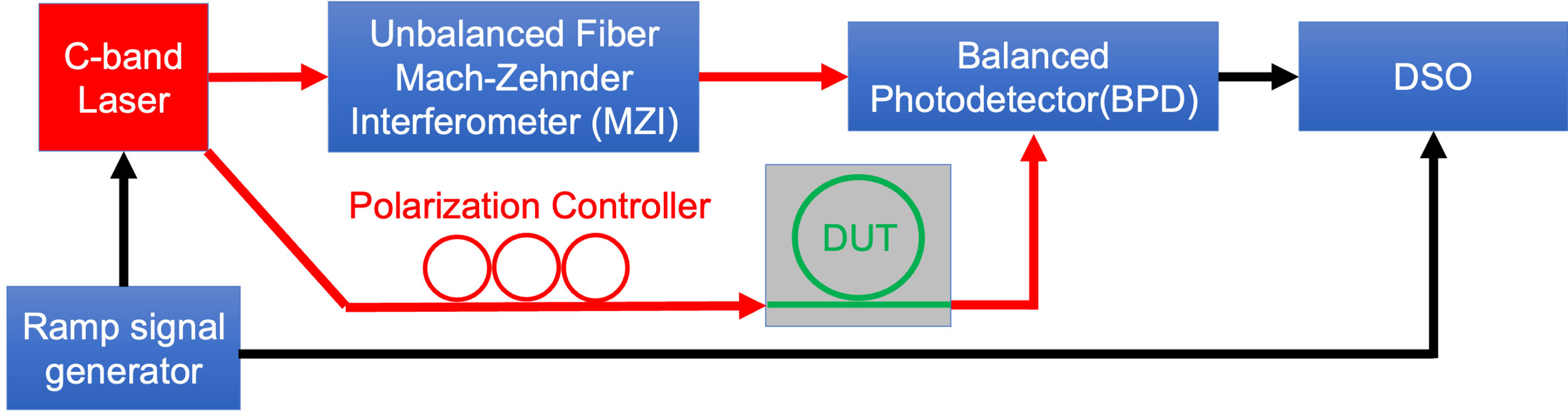


**Fig. S9 Calibrated unbalanced Mach-Zehnder Interferometer (MZI) setup for Q factor measurements.**

The full-width-at-half-maximum resonance width of the single bus ring resonators is measured with the radio frequency calibrated Mach-Zehnder interferometer (MZI) to extract the quality factor. The propagation loss of the waveguide is extracted based on the following equation^6^,


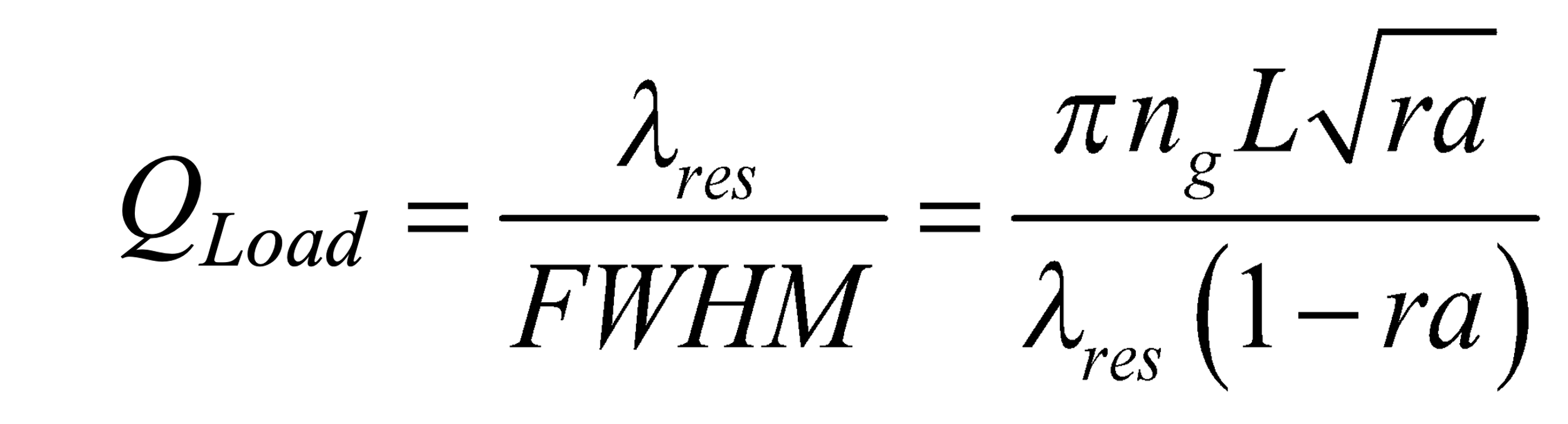
 (ES1)

where *Q_Load_* is the loaded quality factor. $n_{g}$ is the group index of the waveguide, 𝐿 = *2*𝜋*R* is the perimeter of the ring resonator, 𝜆res is the resonant wavelength, $r=\sqrt{1 - \kappa^{2}}$ is the self-coupling coefficient and $\kappa^{2}$ is the power coupling coefficient, *a* is the single-pass amplitude transmission and is related to the power attenuation coefficient 𝛼 as $a^{2}$ = 𝑒𝑥𝑝(-𝛼𝐿). The intrinsic Q of the resonator can be calculated with the extraction of waveguide propagation loss 𝛼 using the following equation^7^.


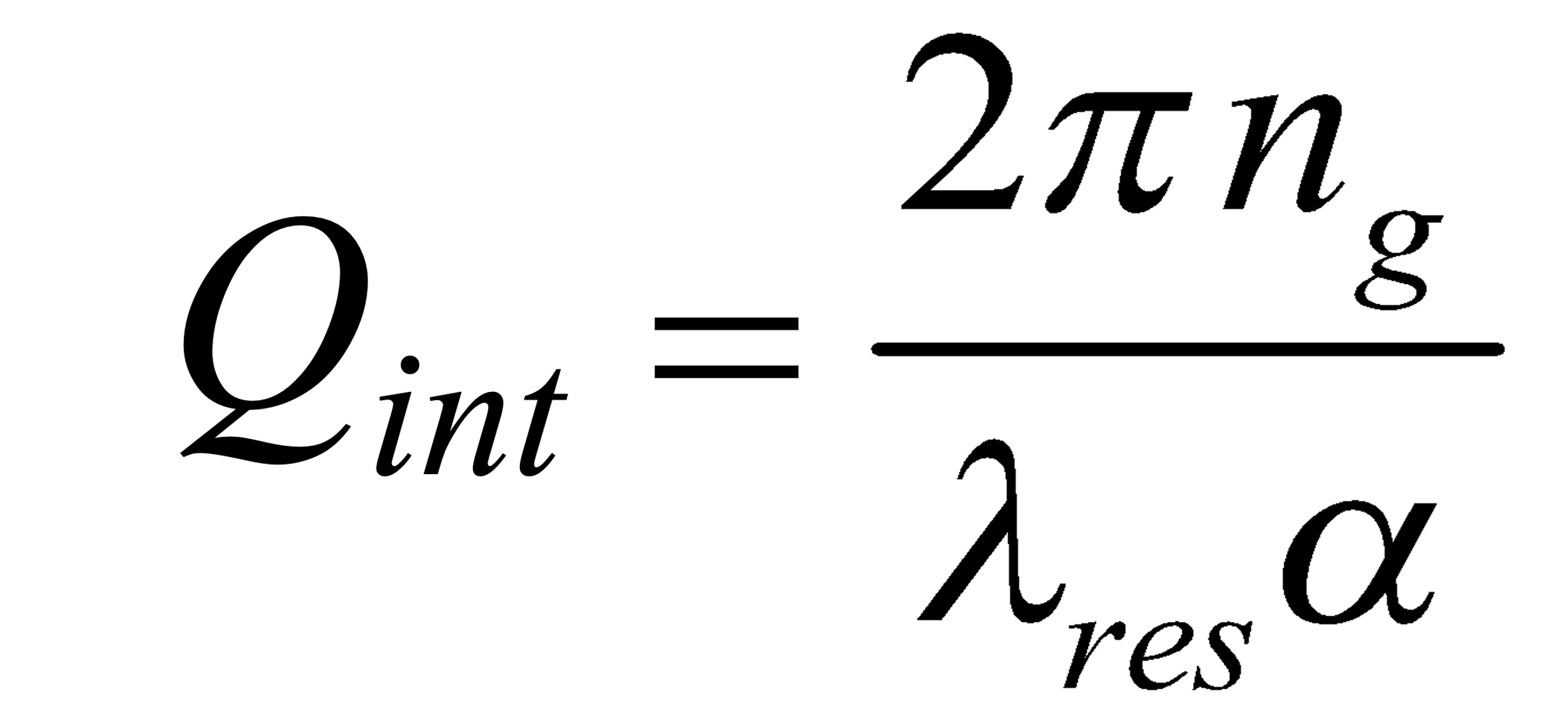
 (ES2)

The group indexes we use for equation ES2 for loss calculations are from Free Spectral Range (FSR) measurements and are 1.4642 for the 80 nm x 6 μm thin nitride TM mode^8^, and 2.025 and 2.053 in the TE and TM modes for the 2 μm wide 800 nm thick devices.

For the ICP-PECVD thin nitride resonators, the TM resonances for all devices below 1550 nm are undercoupled, while almost all resonances 1550 nm and above are overcoupled. As an example to determine whether a TM mode is under or overcoupled, we take the case of our lowest loss 1.77 dB m^-1^ resonance at 1550 nm. The simulated ring-bus field coupling ($\kappa$) using refractive indices from ellipsometry at 1550 nm (Table TS1) and the actual waveguide dimensions (Fig. 3d) using Lumerical FDTD is 0.2141. The undercoupled solution for this resonance gives a k of 0.14, while the overcoupled solution gives a k of 0.2379 which is within the tolerance of our measurement to the simulated value. The overcoupled value of $\kappa$ here also agrees better with measurements of $\kappa$ from ring-bus coupling structures present on the same chip (Fig. 3c). The TE mode resonances for the thin nitrides are all undercoupled, and are difficult to measure accurately to calculate Qi and loss for all wavelengths for all of the devices because of the low extinction of the resonances. For the unannealed Low Pressure Chemical Vapor Deposited (LPCVD) devices in Section S7, the TM modes are all overcoupled.

The resonances for the thick nitride resonators shown are all undercoupled for both the TE and TM modes. These resonances are fit to a modified Lorentzian curve to account for resonance splitting caused by backscattering in the ring^9^. Some statistics about the Qs and losses measured of the 2 μm waveguide width and 300 nm gap thick nitride device is given in Table TS3 below.

**Table TS4. Thick nitride median and average intrinsic Q and losses**

| **Mode** | **Median of intrinsic Qs (millions)** | **Average of intrinsic Qs (millions)** | **Median of losses  (dB m^-1^)** | **Average of losses  (dB m^-1^)** |
| --- | --- | --- | --- | --- |
| TE | 2.59 | 2.60 | 13.9 | 14.8 |
| TM | 1.07 | 1.11 | 32.9 | 34.1 |

The loss and Q of the low temperature thick nitride devices with similar area were calculated using the same split resonance model as we used for our own devices and fitting to the data in the “Fig. 3c” in Y. Xie et al^10^. This resonance had a loaded Q of 1.5 million, at 1560.39 nm, with a Free Spectral Range (FSR) of 150 GHz as given in Y. Xie et al^10^, yielding an intrinsic Q of 2.9 million and loss of 11.9 dB m^-1^.

**S7. Absorption loss estimation**

We measure the absorption loss at 1550 nm of thin nitride device 3 to separate the contributions of absorption and scattering losses to the total loss. This measurement follows a technique to quantify the photothermal induced bistable linewidth shift of the longitudinal ring resonances^9^, using a spectral scan across the resonance with a high on-chip power, to induce a photothermal resonance redshift that is comparable to the resonance linewidth. This photothermal effect is due to absorption heating in the resonator. As shown in Fig. S10a, the red detuning (from shorter to longer wavelengths) across resonance heats up the resonator, and induces a resonance redshift, resulting in a skewed lineshape. To extract the absorption loss relative to the total loss, we simulate the thermal impedance *R_th_* of the ring resonator in Comsol®, giving us *R_th_* = 6.87 K W^-1^. We then measure the thermal-optic redshift with a global heating *Δf_res_/ΔT* = 1.23 GHz K^-1^ which yields the resonance redshift per milliwatt of optical power absorbed by the resonator *Δf_res_/P_abs_* = 8.45 MHz mW^-1^. The resonance redshift has a linear relationship with on-chip power (Fig S10b), confirming the photothermal heating effect, from which we extract the absorption loss fraction to be 59 %. This yields an absorption loss of 1.04 dB m^-1^, which can be said to be an upper bound for the absorption loss of the cladding in this device. Some of this absorption loss might come from the 1520 nm loss peak (See W.Jin et al^1^ and our(Fig. 4a)) due to SiN-H bonds in the nitride due to residual hydrogen, even for thin nitrides.


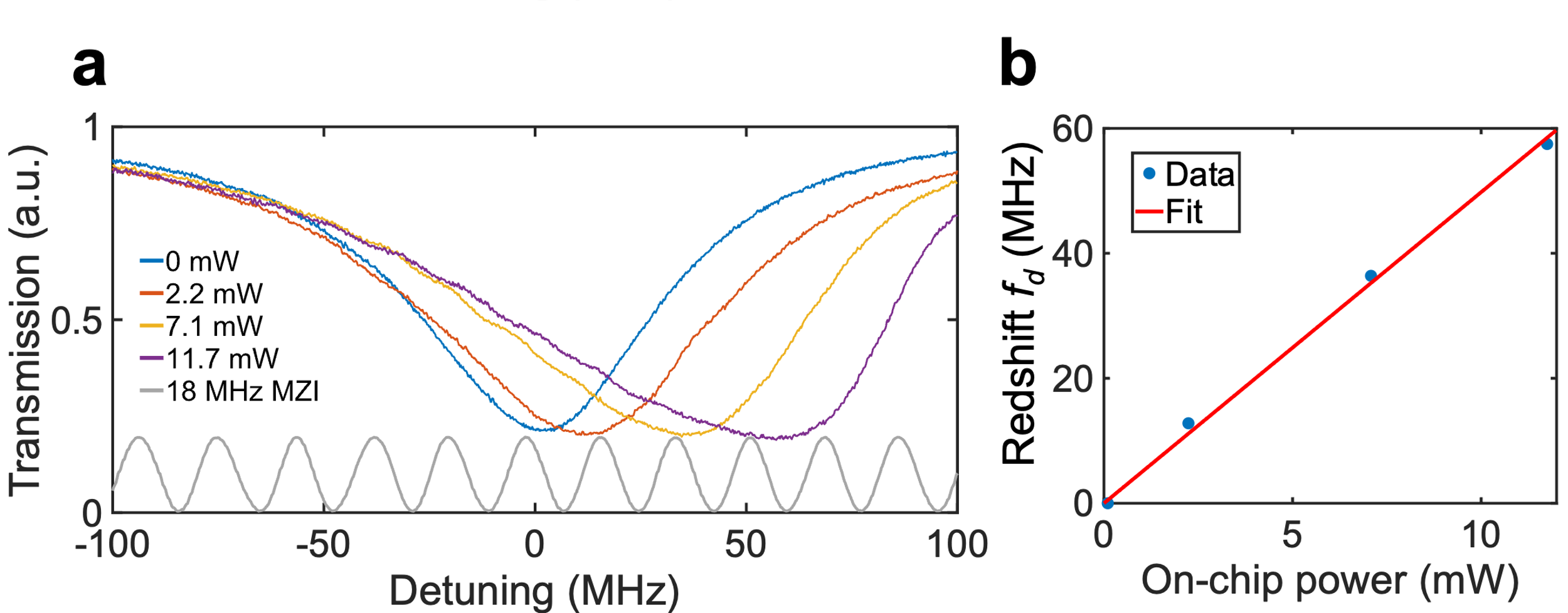


**Fig. S10. Photo-thermal heating and absorption loss measurement.** **a** Photothermal effect is amplified by higher on-chip power and the resonance redshift exhibits a linear relationship with the on-chip power. **b** Normal Lorentzian fitting for the lower power spectral sweeping and skewed Lorentzian fitting for the high power spectral sweeping extracts the intrinsic loss and absorption loss rates.

**S8. Thermorefractive Noise (TRN) Floor Estimation, PDH locking and frequency noise measurements**

From the modal area (Table TS3) and cavity length of a device, one can estimate the thermorefractive noise (TRN) floor of said cavity^11^. Our thin nitride cavity is hence estimated to have a TRN floor of around 10 Hz^2^ Hz^-1^ which is more than 3 orders of magnitude lower than the TRN floor for a typical thick nitride ring resonator enabling us to lower the frequency noise of a locked laser to a cavity by 3 orders of magnitude more than would be possible with a typical thick nitride device.

**Table TS5. TRN Floor Estimation for different device designs**

| **Device** | **Cavity Length** | **Mode Area** | **TRN Floor at 10^4^ Hz offset** |
| --- | --- | --- | --- |
| K. Liu et al^11^ | 4 m | 18 um^2^ | 0.1 Hz^2^ Hz^-1^ |
| Typical thick nitride | 1 mm | 1 um^2^ | 14,250 Hz^2^ Hz^-1^ |
| Our thin cavity | 53.6 mm | 27 um^2^ | 9.85 Hz^2^ Hz^-1^ |

In the case where one makes a thick nitride resonator with as long a length as the thin nitride mode size is larger (27 times), one will still not be able to reach as low frequency noise when locked as the same also depends inversely on the quality factor^11^. Further, increasing a thick nitride resonator length to 1.5 m would increase the waveguide loss typically due to increase in the total number of accumulated defects in the resonator waveguide, further increasing the lowest frequency noise possible; we see at least 2x increase in losses between resonators of lengths similar to the thin nitride resonator in this work, and resonators several meters in length in waveguides of similar geometries - in our other works.

Laser frequency stabilization was achieved using a standard Pound Drever Hall locking arrangement, as shown in Fig. 4c, and described in detail in K. Liu et al^11^. A velocity TLB 6730-P tunable External Cavity Diode Laser (ECDL) was used as the laser source, with 30 MHz phase modulated sidebands applied via current modulation. Approximately 1 mW optical power was delivered to the resonator, and the resulting transmission signal was photodetected with a 10 kV A^-1^ Trans-Inductance Amplifier (TIA) amplified photodetector.


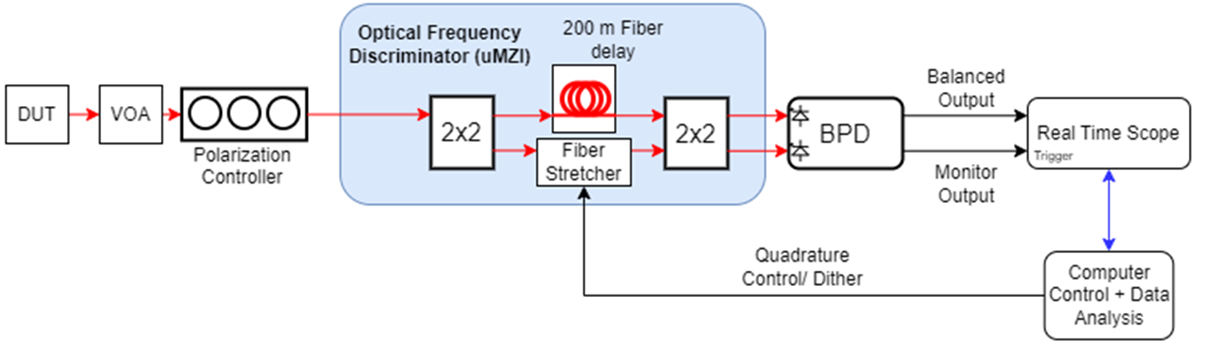


**Fig. S11 Detailed system diagram for laser frequency noise measurement using an optical frequency discriminator.**

Frequency noise measurements were carried out using an unbalanced Mach-Zender interferometer with a 200 m delay length as in Fig. S11 above, and described in detail in G. Brodnik et al^12^.

The low frequency cutoff for the linewidth calculation was 300 Hz, and the high frequency cutoff was 990,000 Hz.

**S9. Thin nitride loss comparison with LPCVD nitride**

We compare the losses of devices made using the thin nitride geometry (80 nm x 6 μm) between those using deuterated ICP-PECVD nitride cores to those using unannealed Low Pressure Chemical Vapor Deposited (LPCVD) cores, both using the same deuterated upper cladding, for a fair comparison. We see that at 1550 nm and above, the losses are very much comparable.

**
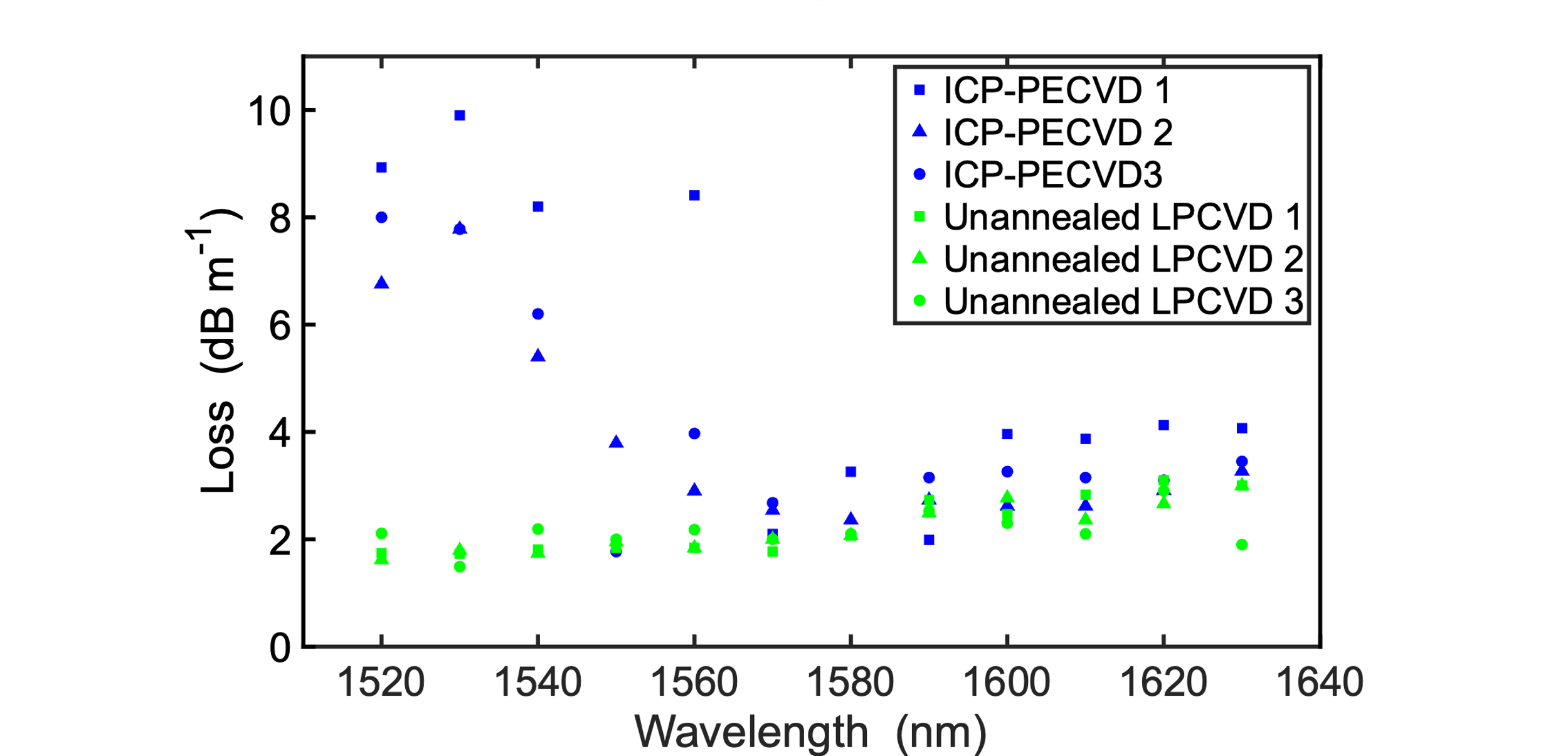
**

**Fig. S12 Loss comparison between thin nitride devices made using different processes.** The losses of our 250 °C Inductively Coupled Plasma-Plasma Enhanced Chemical Vapor Deposition (ICP-PECVD) process devices are similar to those made using unannealed Low Pressure Chemical Vapor Deposition (LPCVD) nitride cores, both using the same deuterated upper cladding.

**S10. Additional Q/loss measurements of thick nitride devices**

Additional Q measurements of the thick nitride TE modes for the 175 μm radius device around the wavelengths where the loss minima or outliers in wavelength occur in Fig. 5a are taken (Fig. S13) namely around 1580, 1600, and 1606 nm respectively, showing that these are not measurement discrepancies and may be related to avoided mode crossings with higher order modes.


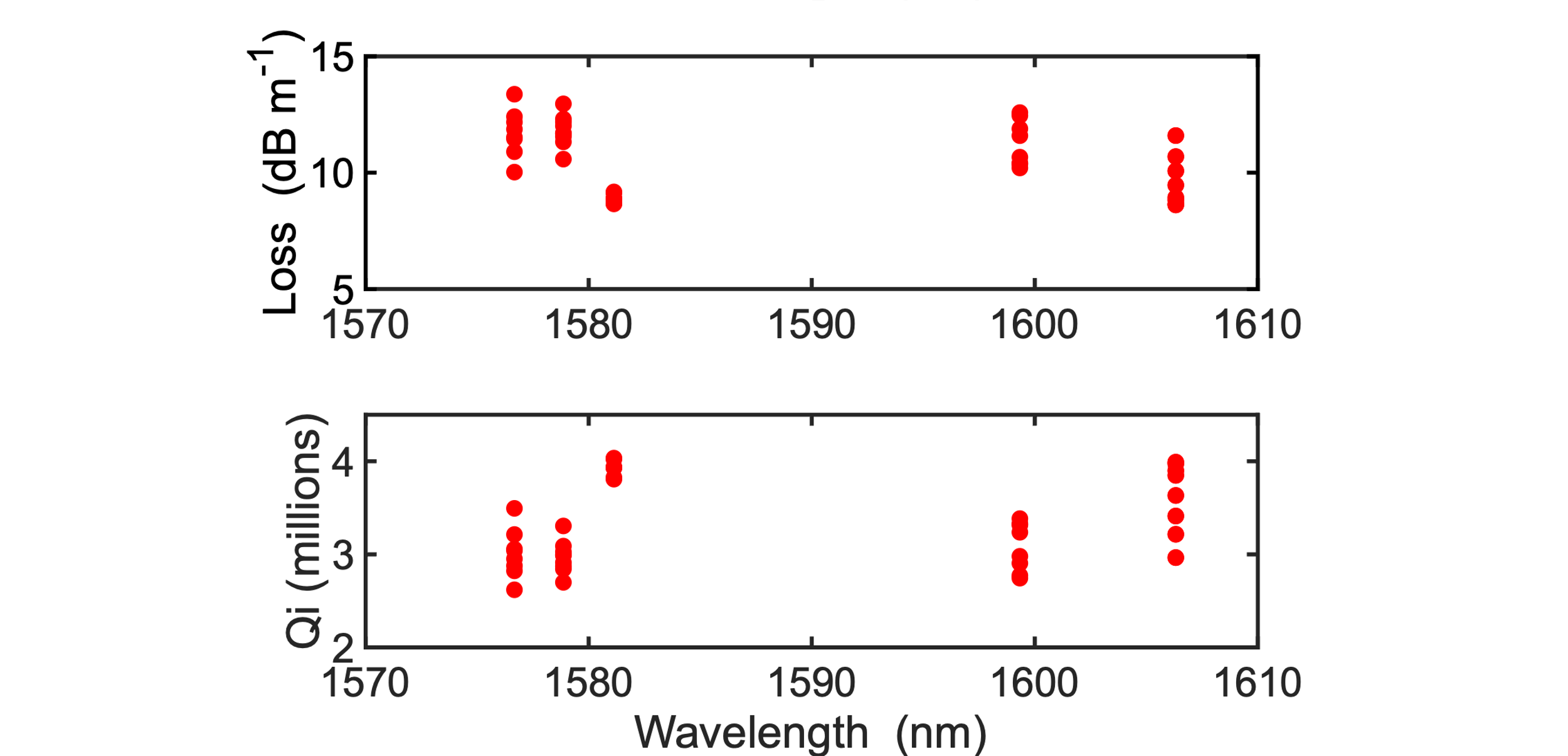


**Fig. S13 Additional Q/loss measurements near loss minima.** Additional Q/loss measurements were taken around 1580, 1600, and 1606 nm respectively showing consistency of the Q/loss values between measurements.

We also take more Q/loss measurements of 3 different ring resonator devices with 165 μm radii, but also having a 300 nm ring-bus gap and 2 μm wide waveguide similar to the devices in Fig. 5, for the TE modes. The losses and Qs being fairly consistent across the different devices shows that our process is reliable (Fig. S14).

**
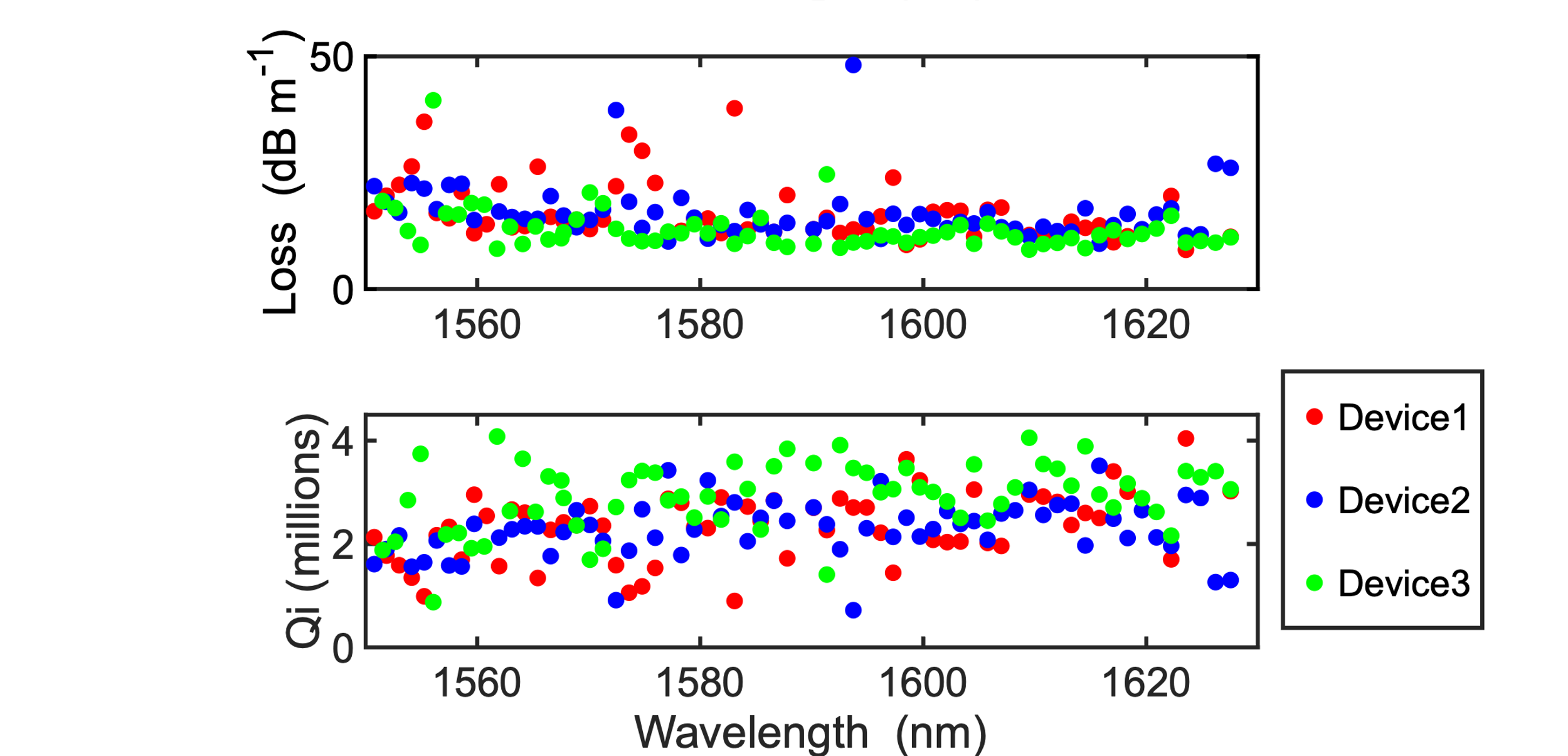
**

**Fig. S14 Loss and Q comparison between 3 different thick nitride ring resonator devices of radius 165 um, 2 um wide waveguide, with 300 nm ring-bus gaps for the TE modes.** Our loss and intrinsic Qs are fairly consistent across the different devices

The median and average losses for TE modes for these devices is given in Table TS6 below.

**Table TS6. Median and average intrinsic Q and losses for the three 165 μm ring resonator devices**

| **Mode** | **Median of intrinsic Qs (millions)** | **Average of intrinsic Qs (millions)** | **Median of losses (dB m^-1^)** | **Average of losses (dB m^-1^)** |
| --- | --- | --- | --- | --- |
| **TE** | 2.51 | 2.50 | 13.7 | 19.2 |

The loss in a 0.35 m long spiral of the 2 um wide thick waveguide for the TE mode was also measured using Optical Backscatter Reflectometry (OBR) between 1535 to 1600 nm wavelengths as in Fig. S15 below, and reaches almost 15 dB m^-1^ at the longer wavelengths. This loss is slightly higher than that measured using ring resonators as the longer length causes more overall waveguide defects and/or particles to accumulate near the waveguide and results in a higher scattering loss.^13^


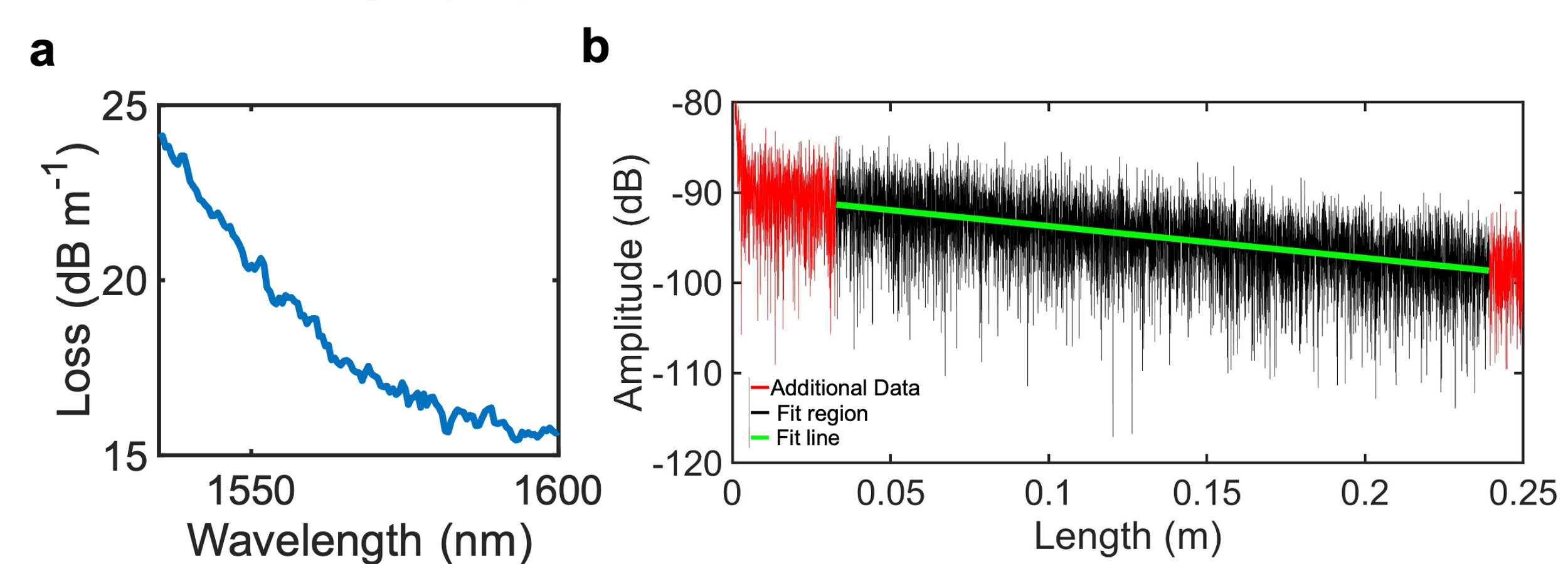


**Fig. S15 Optical Backscatter Reflectometry (OBR) Measurements on a 0.35 m long spiral of a core geometry of 0.8 x 2 um. a** Loss vs wavelength of the spiral between 1535 to 1600 nm wavelengths. The loss is lower at the longer wavelengths and goes down to almost 15 dB m^-1^. **b** Backscattered power vs length at 1600 nm for the spiral. We fit the data for a length of around 0.2 m to extract the loss.

**S11. Calculations and additional non-linear application measurements of thick nitride devices**

The Optical Parametric Oscillation (OPO) thresholds and thresholds per unit length of various works are calculated and shown below in Table TS7.

**Table TS7. OPO Threshold per unit length of different works**

| **OPO threshold (mW)** | **Ring resonator radius (μm)** | **OPO threshold per unit length (mW mm^-1^)** | **Work** |
| --- | --- | --- | --- |
| 16.7 | 175 | 15.2 | This work. |
| 23.7 | 160 | 23.6 | Y. Xie et al^10^ |
| 13.5 | 80 | 26.9 | Z. Wu et al^3^ |
| 40 | 23 | 276.8 | J. Chiles et al^14^ |
| 10 | 50 | 31.8 | X.X. Chia et al^15^ |
| 21 | 100 | 33.42 | Z. Ye et al^16^ |

Kerr comb formation was measured using a widely tunable ECDL amplified by a high power EDFA. The laser frequency was tuned to be slightly blue detuned from a TE mode resonance located at 1566.7 nm (at low optical power), and the resonator output was monitored with an optical spectrum analyzer. As optical power was increased, the laser frequency was slowly tuned to maintain the smallest possible blue detuning between laser and resonator. On-chip power was calculated by subtracting half the total throughput coupling loss of the resonator from the measured input optical power to the chip. At on-chip powers higher than 40 mW, The comb transitions into the modulation instability regime, as seen in Figure S16 below. Due to difficulties with strong thermal shifting and a large number of avoided mode crossings, soliton steps were not able to be observed.


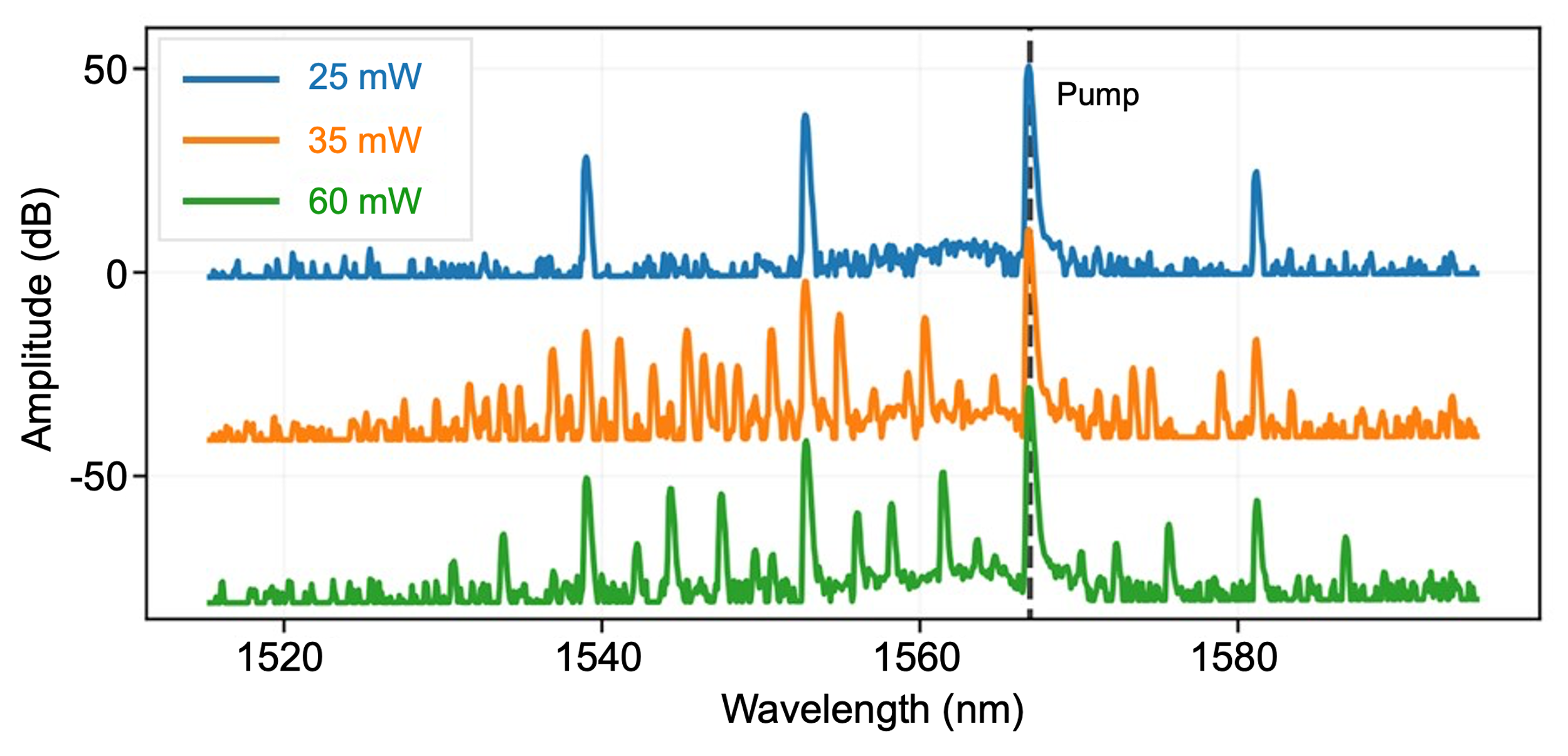


**Fig. S16 Kerr comb formation.** Thick nitride Kerr comb evolution at various on-chip pump powers.

To confirm that the device is able to support soliton formation, a temporal Lugiato-Lefever equation simulation^16^ was used to simulate soliton formation dynamics of the 2000 nm TE mode ring-resonator, using dispersion information measured (Fig. S17 (b)) via a widely tunable continuous laser sweep. Figure S17 (a) below shows a laser detuning sweep with 600 mW on-chip power that results in multiple soliton steps.


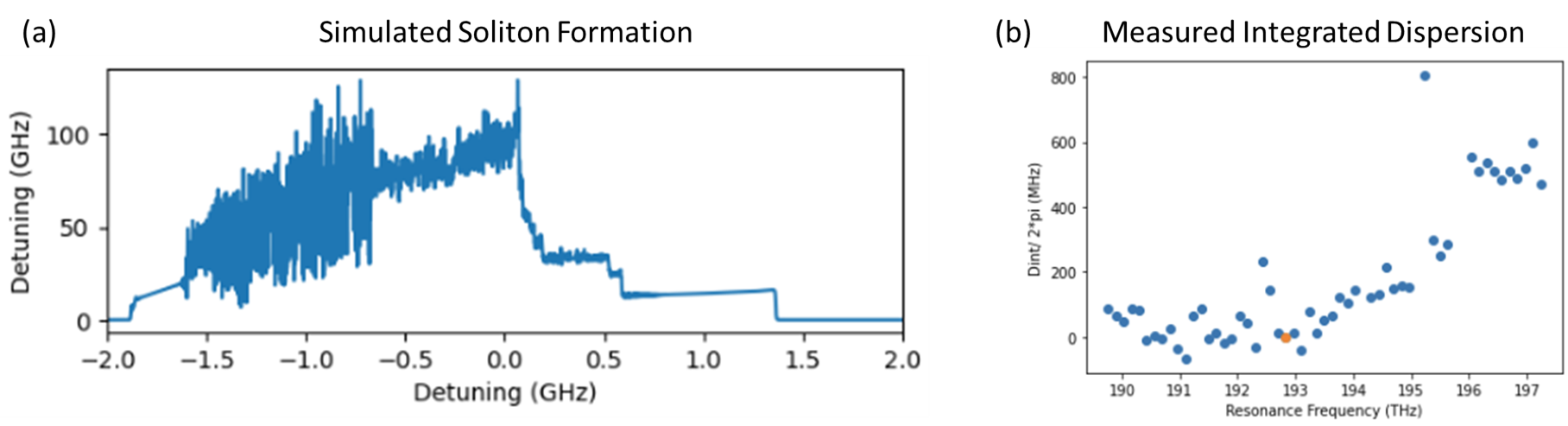
**Fig. S17 (a)**  Simulated Intra-cavity power versus laser-resonator detuning at 600 mW pump power on chip. Multiple step-like transitions demonstrate the existence of multiple soliton states at high pump powers. **(b)** Integrated dispersion for the TE mode of the 2000 nm thick resonator, showing significant variance in FSR, as well as some strong avoided mode crossings.

**S12. Comparison table of losses/Qs between different processes**

Below is a comparison table of losses/Qs of nitride ring resonators from previous works made using different processes that we used to make Fig. 7.

**Table TS8. Loss/Q of ring resonators made with different processes**

| **Work** | **Nitride Growth Process** | **CT (nm)** | **RWW (um)** | **MPT (°C)** | **Loss (minimum)** | **Intrinsic Q (maximum)** | **Other comments** |
| --- | --- | --- | --- | --- | --- | --- | --- |
| This work | ICP-PECVD (Deuterated) | 80 800 | 6 2 | 250 | 1.77 dB m^-1^ 8.66 dB m^-1^ | 14.9 million 4.03 million |  |
| Y. Xie et al^10^ | ICP-PECVD (Deuterated) | 850 | 2.2 | 270 | 6 dB m^-1^ 11.9 dB m^-1^ | 5.3 million 2.9 million | 480 um radius 160 um radius (latter calculated) |
| J. Chiles et al^14^ | ICP-PECVD (deuterated) | 920 | 2.3 | 270 | 22 dB m^-1^ | 1.6 million | Only 430 nm of nitride etched |
| X. X Chia et al^15^ | ICP-PECVD (deuterated) | 320 | 1.2 | 350 | 320 dB m^-1^ | 135 thousand |  |
| A. Frigg et al^17^ | Sputtering | 850 | 1.8 | 400 | 50 dB m^-1^ | 0.66 million |  |
| S. Zhang et al^18^ | Sputtering | 750 | 1.8 | 300 400 | 32 dB m^-1^ 5.4 dB m^-1^ | 1.1 million 6.2 million | subsequent anneal |
| X. Ji et al^19^ | PECVD + CMP | 730 | 1.5 | 350 | 42 dB m^-1^ | 0.72 million |  |
| N. Golshani et al^20^ | Pulsed Laser Deposition | 840 | 0.4 | 400 | 25 dB m^-1^ | 1.43 million | Q calculated |
| Z. Ye et al^21^ | LPCVD | 810 | 2.4 | 1200 | 2.6 dB m^-1^ | 14 million |  |
| X. Ji et al^22^ | LPCVD | 730 | 10 | 1200 | 0.4 dB m^-1^ | 67 million |  |
| K. Liu et al^23^ | LPCVD | 80 | 11 | 1150 | 0.034 dB m^-1^ | 720 million |  |
| J. Liu et al^13^ | LPCVD | 950 | 2.2 | 1250 | 1 dB m^-1^ | 30 million | Most probable value only mentioned in work |
| W. Sun et al^24^ | LPCVD | 300 | 2.6 | 1200 | 0.77 dB m^-1^ | 42 million | Loss calculated. |

CT = Nitride Core Thickness, WW = Ring Waveguide Width, MPT = Maximum Process Temperature; All the LPCVD works used high temperature annealing.

**Part 1 References**

1. Jin, W. *et al.* Deuterated silicon dioxide for heterogeneous integration of ultra-low-loss waveguides. *Opt. Lett., OL* **45**, 3340–3343 (2020).

2. UCSB Nanofabrication Facility. PECVD Recipes - UCSB Nanofab Wiki. https://wiki.nanofab.ucsb.edu/wiki/PECVD_Recipes#ICP-PECVD_.28Unaxis_VLR.29.

3. Wu, Z. *et al.* Low-noise Kerr frequency comb generation with low temperature deuterated silicon nitride waveguides. *Opt. Express, OE* **29**, 29557–29566 (2021).

4. Dergez, D., Schalko, J., Bittner, A. & Schmid, U. Fundamental properties of a-SiNx:H thin films deposited by ICP-PECVD for MEMS applications. *Applied Surface Science* **284**, 348–353 (2013).

5. Gundavarapu, S. *et al.* Sub-hertz fundamental linewidth photonic integrated Brillouin laser. *Nature Photon* **13**, 60–67 (2019).

6. Bogaerts, W. *et al.* Silicon microring resonators. *Laser & Photonics Reviews* **6**, 47–73 (2012).

7. Chrostowski, L. & Hochberg, M. *Silicon Photonics Design: From Devices to Systems*. (Cambridge University Press, Cambridge, 2015). doi:10.1017/CBO9781316084168.

8. Bose, D., Wang, J. & Blumenthal, D. J. 250C Process for < 2dB/m Ultra-Low Loss Silicon Nitride Integrated Photonic Waveguides. in *Conference on Lasers and Electro-Optics (2022), paper SF3O.1* SF3O.1 (Optica Publishing Group, 2022). doi:10.1364/CLEO_SI.2022.SF3O.1.

9. Puckett, M. W. *et al.* 422 Million intrinsic quality factor planar integrated all-waveguide resonator with sub-MHz linewidth. *Nat Commun* **12**, 934 (2021).

10. Xie, Y. *et al.* Soliton frequency comb generation in CMOS-compatible silicon nitride microresonators. *Photon. Res., PRJ* **10**, 1290–1296 (2022).

11. Liu, K. *et al.* 36 Hz integral linewidth laser based on a photonic integrated 4.0 m coil resonator. *Optica, OPTICA* **9**, 770–775 (2022).

12. Brodnik, G. M. *et al.* Optically synchronized fibre links using spectrally pure chip-scale lasers. *Nat. Photon.* **15**, 588–593 (2021).

13. Liu, J. *et al.* High-yield, wafer-scale fabrication of ultralow-loss, dispersion-engineered silicon nitride photonic circuits. *Nat Commun* **12**, 2236 (2021).

14. Chiles, J. *et al.* Deuterated silicon nitride photonic devices for broadband optical frequency comb generation. *Opt. Lett., OL* **43**, 1527–1530 (2018).

15. Chia, X. X. *et al.* Low-Power Four-Wave Mixing in Deuterated Silicon-Rich Nitride Ring Resonators. *Journal of Lightwave Technology* **41**, 3115–3130 (2023).

16. Ye, Z., Fülöp, A., Helgason, Ó. B., Andrekson, P. A. & Torres-Company, V. Low-loss high-Q silicon-rich silicon nitride microresonators for Kerr nonlinear optics. *Opt. Lett., OL* **44**, 3326–3329 (2019).

17. Frigg, A. *et al.* Optical frequency comb generation using low stress CMOS compatible reactive sputtered silicon nitride waveguides. in *Integrated Photonics Platforms: Fundamental Research, Manufacturing and Applications* vol. 11364 72–79 (SPIE, 2020).

18. Zhang, S. *et al.* Low-Temperature Sputtered Ultralow-Loss Silicon Nitride for Hybrid Photonic Integration. *Laser & Photonics Reviews* 2300642 doi:10.1002/lpor.202300642.

19. Ji, X. *et al.* Ultra-Low-Loss Silicon Nitride Photonics Based on Deposited Films Compatible with Foundries. *Laser & Photonics Reviews* **17**, 2200544 (2023).

20. Golshani, N. *et al.* Low-loss, low-temperature PVD SiN waveguides. in *2021 IEEE 17th International Conference on Group IV Photonics (GFP)* 1–2 (2021). doi:10.1109/GFP51802.2021.9673874.

21. Ye, Z. *et al.* Foundry manufacturing of tight-confinement, dispersion-engineered, ultralow-loss silicon nitride photonic integrated circuits. *Photon. Res., PRJ* **11**, 558–568 (2023).

22. Ji, X. *et al.* Ultra-low-loss on-chip resonators with sub-milliwatt parametric oscillation threshold. *Optica, OPTICA* **4**, 619–624 (2017).

23. Liu, K. *et al.* Ultralow 0.034 dB/m loss wafer-scale integrated photonics realizing 720 million Q and 380 μW threshold Brillouin lasing. *Opt. Lett., OL* **47**, 1855–1858 (2022).

24. Sun, W. *et al.* A chip-integrated comb-based microwave oscillator. Preprint at https://doi.org/10.48550/arXiv.2403.02828 (2024).

25. Moille, G. , Li, Q. , Lu, X. & Srinivasan, K. , pyLLE: a Fast and User Friendly Lugiato-Lefever Equation Solver, *Journal of Research (NIST JRES)*, National Institute of Standards and Technology, Gaithersburg, MD, (2019), https://doi.org/10.6028/jres.124.012

**P2.**

**Supplementary : Resonance Measurement Summary**

Contents

1. Thin Nitride Plot Summary - 80 nm thin nitride ICP-PECVD Transverse Magnetic TM mode measurements by device.
2. Thick Nitride Plot Summary - 800 nm thick nitride measurements by mode for 175 um radius ring resonators with 2 um coupling gaps

1. Thin Nitride Plot Summary

Group index used = 1.4642
(no split mode resonance models are used for fitting)


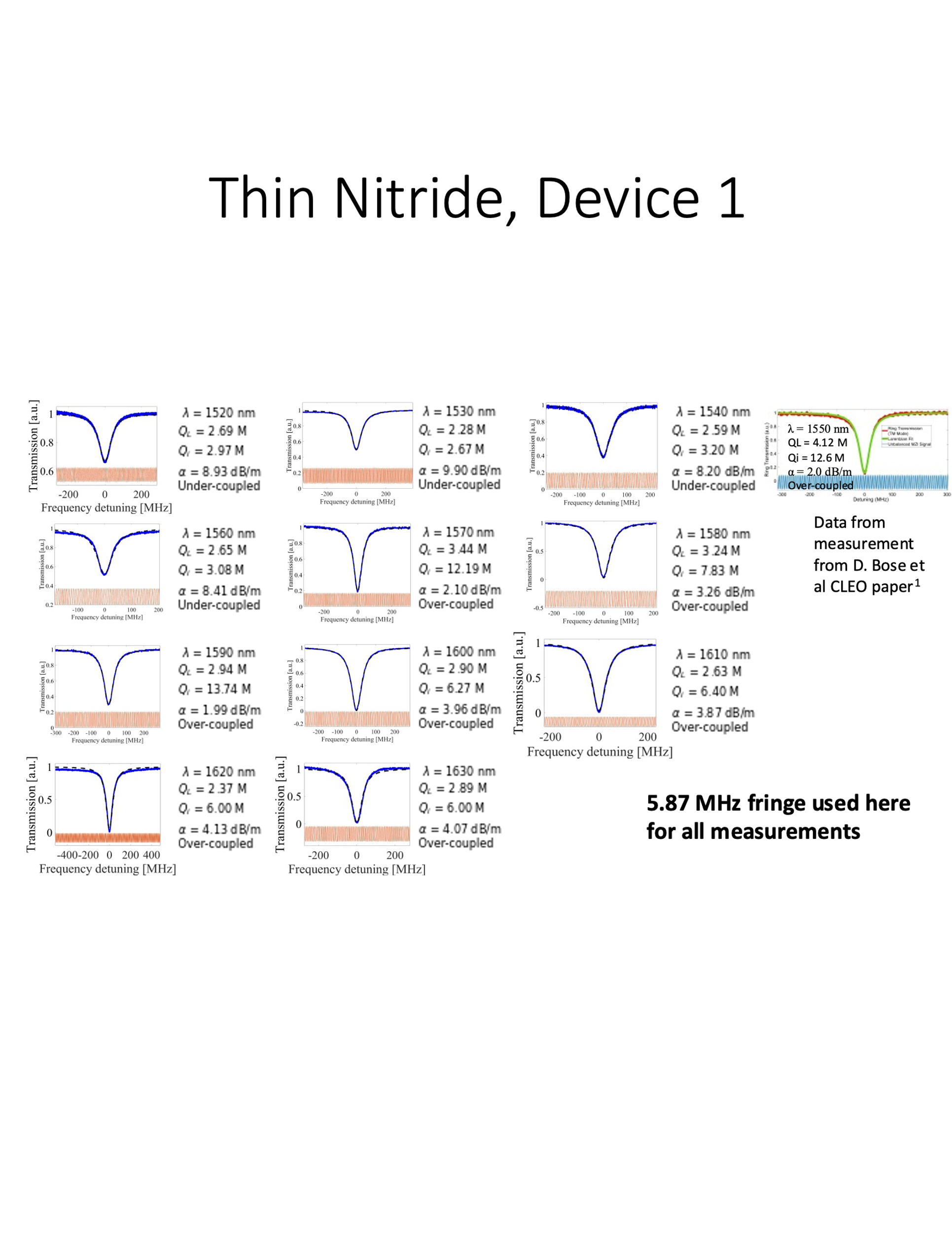


**Fig. S18. Thin nitride, device 1 measurements**


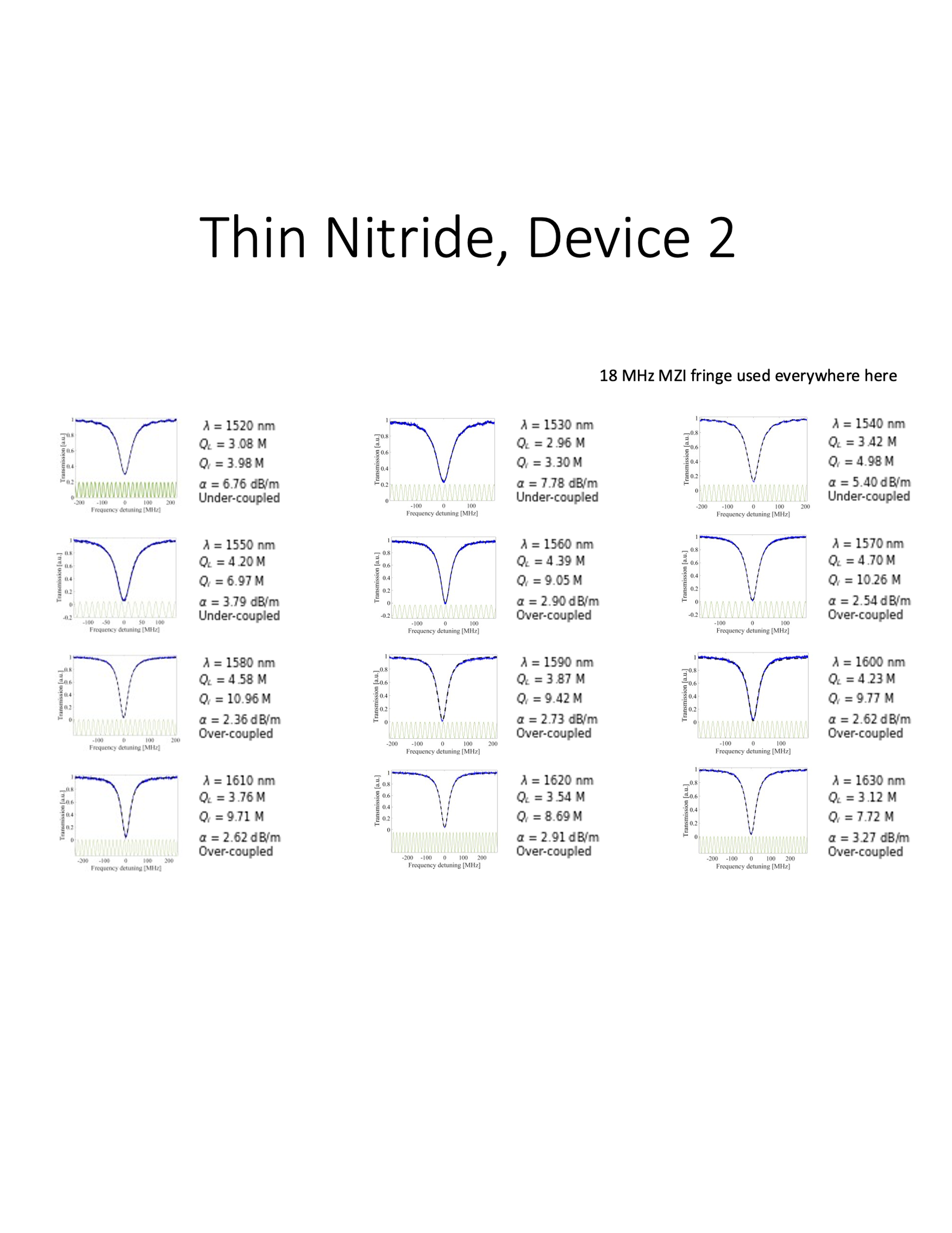


**Fig. S19. Thin nitride, device 2 measurements**


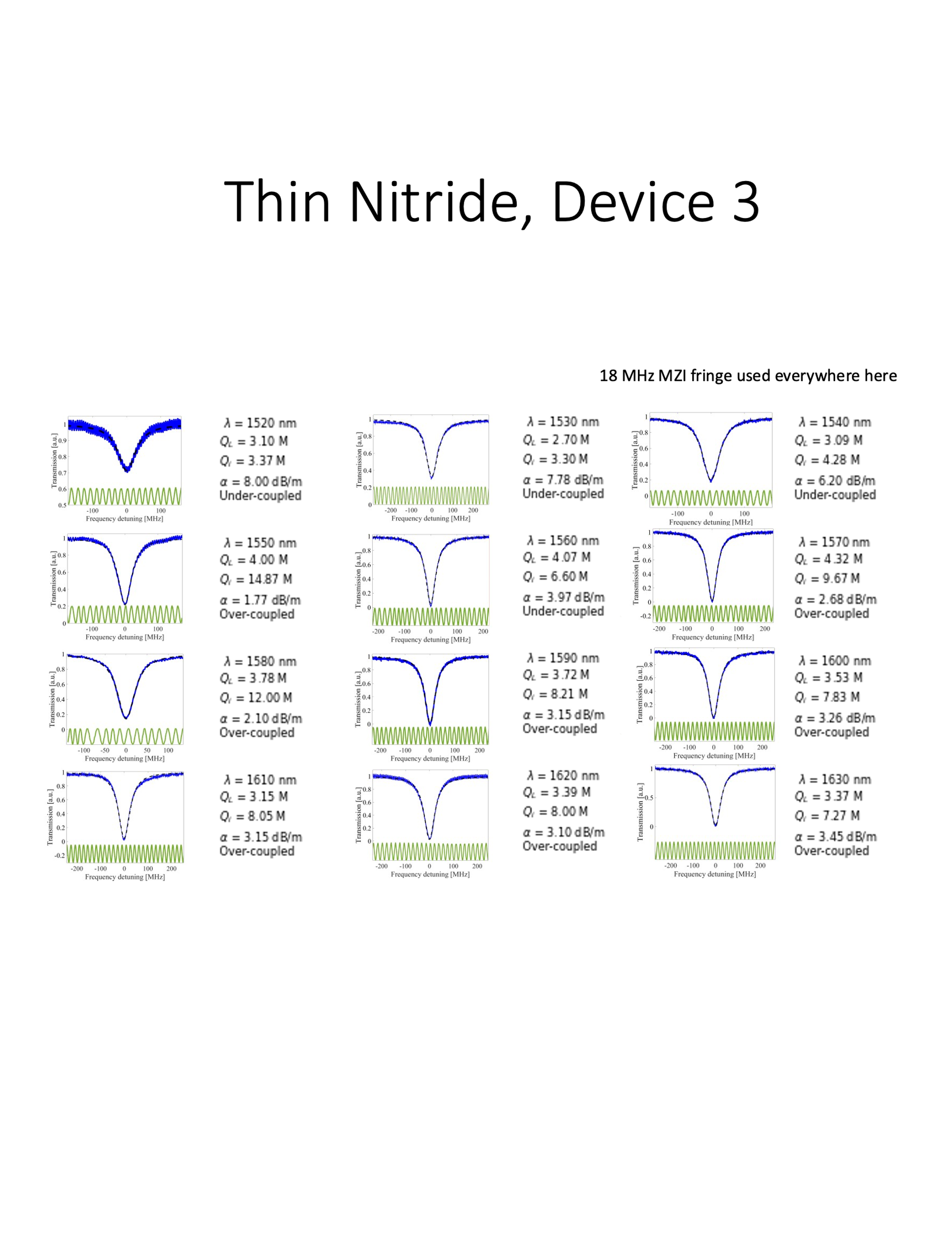


**Fig. S20. Thin nitride, device 3 measurements.**

2. Thick Nitride Plot Summary

Group index used = 2.025 for TE mode, 2.053 for TM mode.

(split mode resonance models are used for fitting)


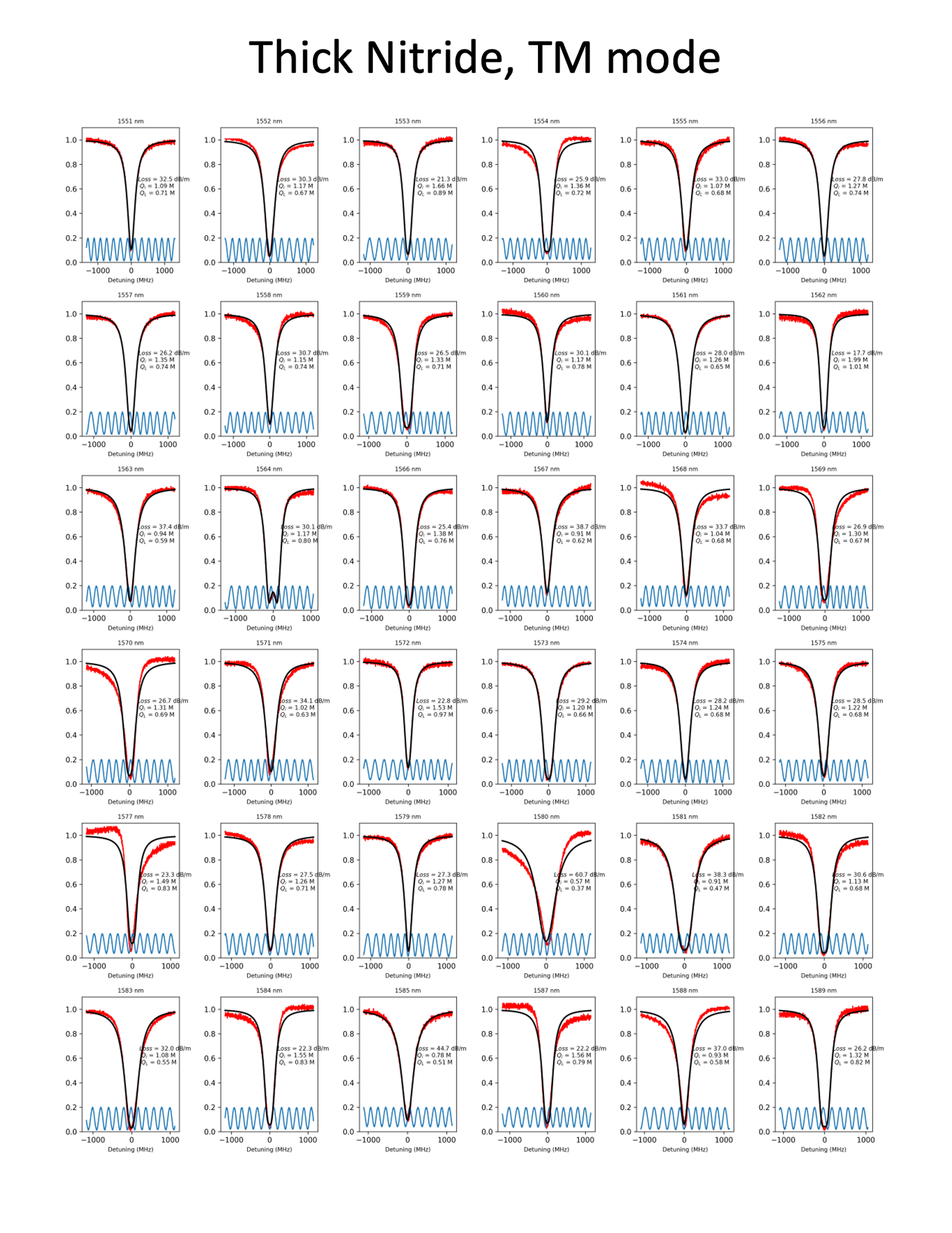


**Fig. S21. Thick nitride Transverse Magnetic (TM) mode measurements.**


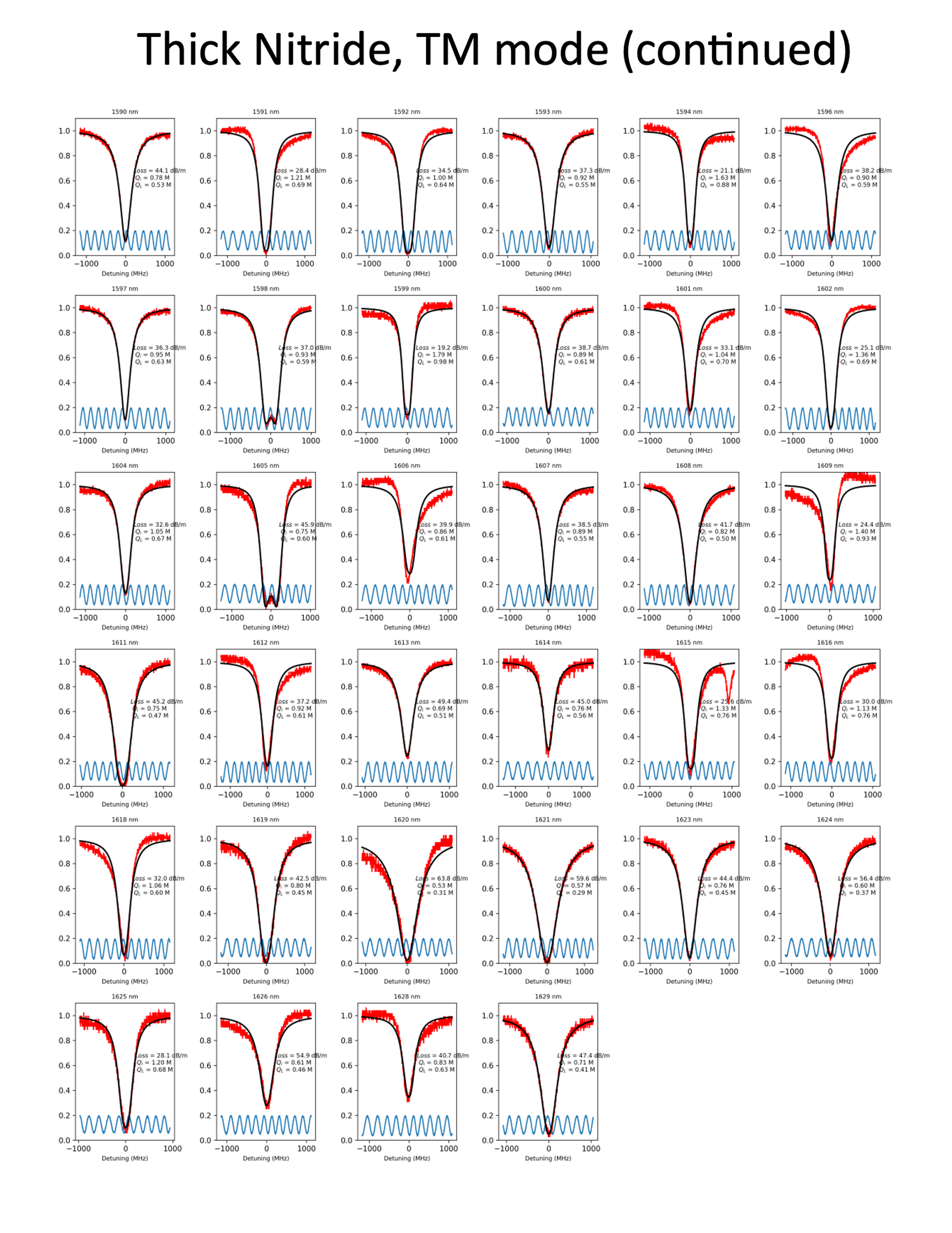
 **Fig. S22. Thick nitride Transverse Magnetic (TM) mode measurements (continued).
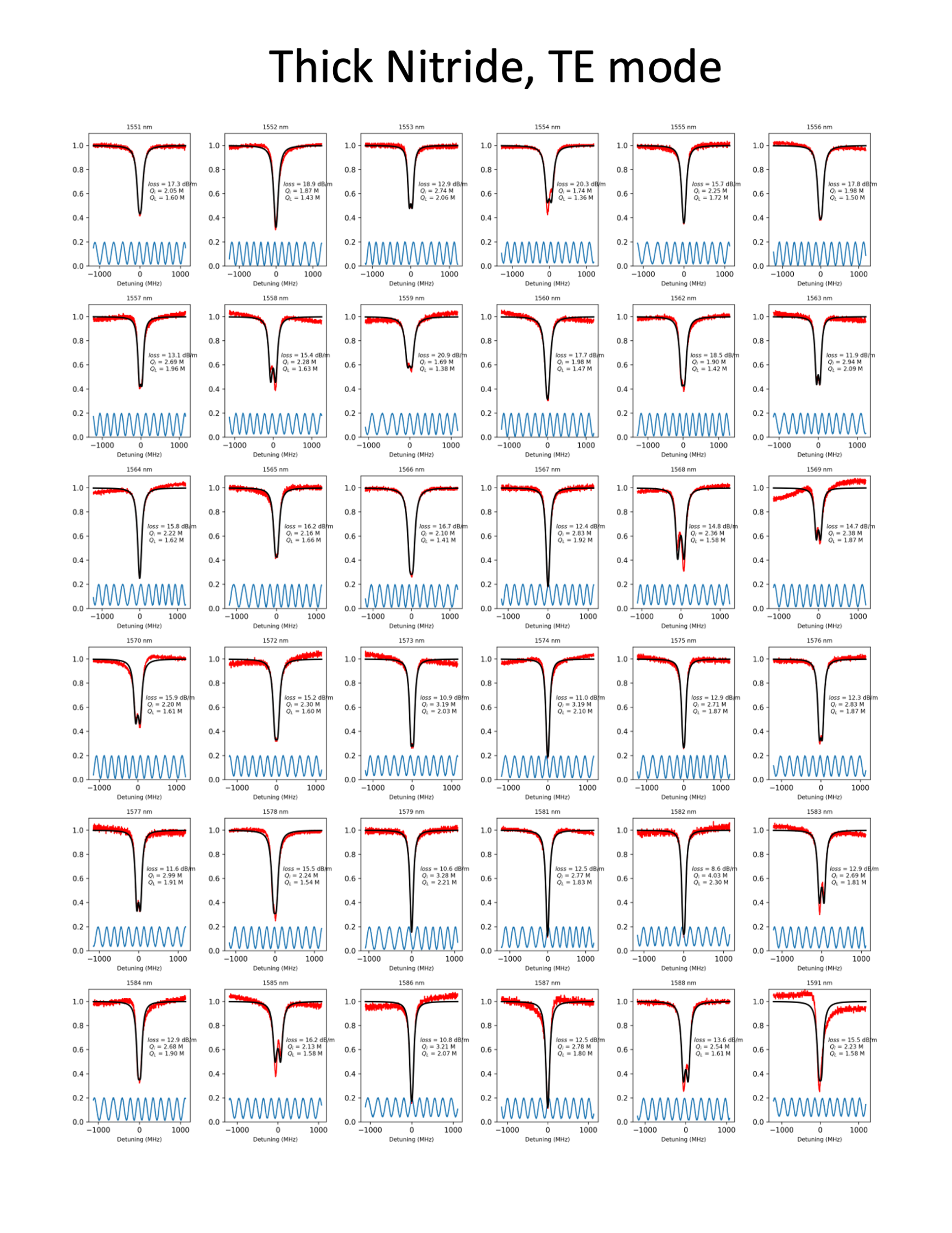
**

**Fig. S23. Thick nitride Transverse Electric (TE) mode measurements**

**
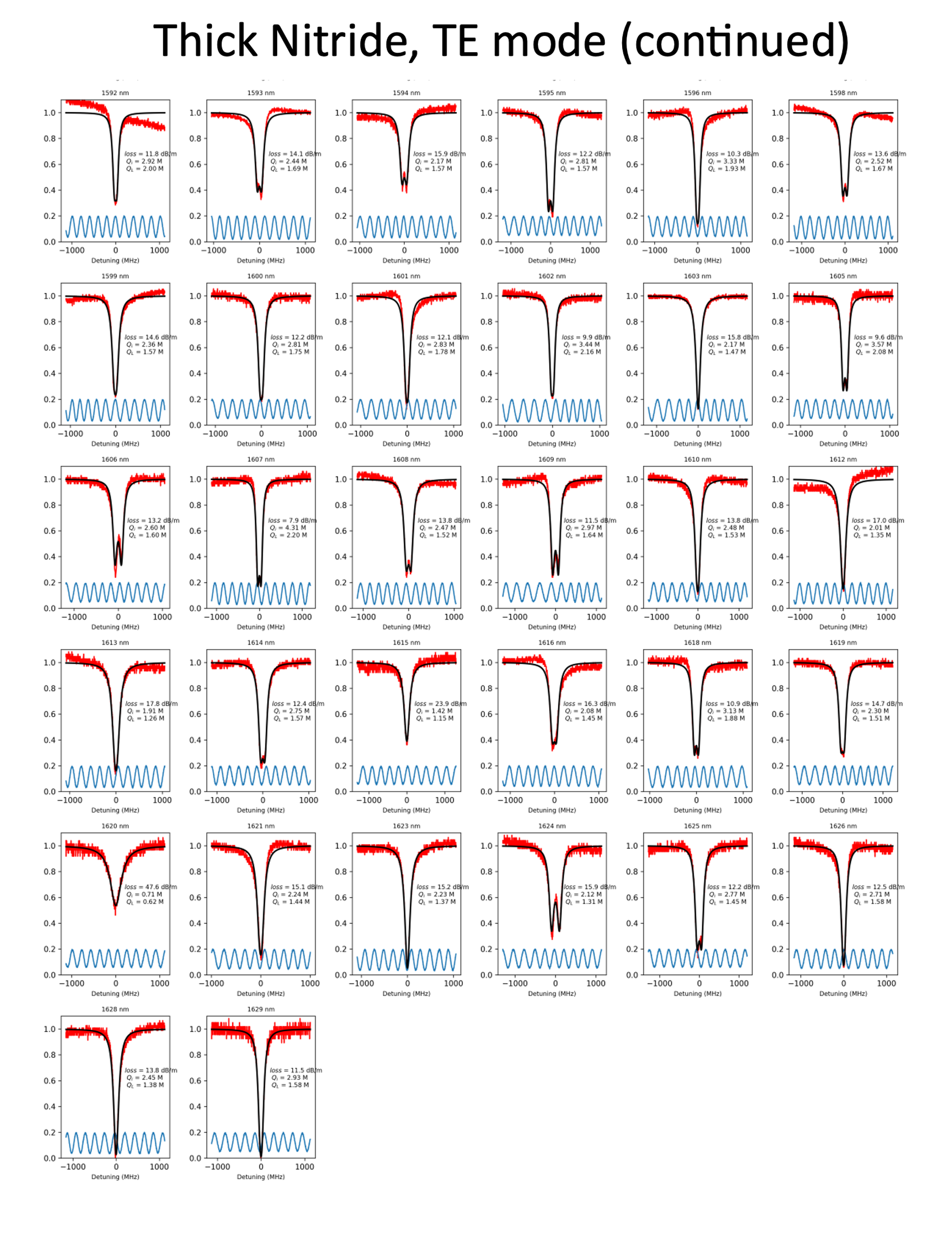
**

**Fig. S24. Thick nitride Transverse Electric (TE) mode measurements (continued)**

**Part 2 References**

1. Bose, D., Wang, J. & Blumenthal, D. J. 250C Process for < 2dB/m Ultra-Low Loss Silicon Nitride Integrated Photonic Waveguides. in *Conference on Lasers and Electro-Optics (2022), paper SF3O.1 SF3O.1* (Optica Publishing Group, 2022). doi:10.1364/CLEO_SI.2022.SF3O.1
